# NSCLC brain metastases exhibit reduced HLA-I antigen presentation machinery and immune evasion independent of IFNγ signaling defects

**DOI:** 10.1186/s12943-026-02687-6

**Published:** 2026-05-28

**Authors:** Noelia Vilariño, Miguel Lopez de Rodas, Maria Villalba-Esparza, Barani Kumar Rajendran, Boyu Huang, Sara Hijazo-Pechero, Adrien Costantini, Kishu Ranjan, Javier Ramos-Paradas, Benjamin Y. Lu, Ernest Nadal, Sarah B. Goldberg, Don X. Nguyen, Kurt A. Schalper

**Affiliations:** 1 Department of Pathology, Yale School of Medicine, New Haven, CT USA; 2 Department of Medical Oncology, Catalan Institute of Oncology (ICO), L’Hospitalet de Llobregat, Barcelona, Spain; 3 Preclinical and Experimental Research in Thoracic Tumors, Oncobell, Bellvitge Biomedical Research Institute (IDIBELL), L’Hospitalet de Llobregat, Barcelona, Spain; 4 Universitat de Barcelona (UB), Barcelona, Spain; 5 Department of Respiratory Diseases and Thoracic Oncology, Cancer Institute APHP. Paris-Saclay University, Hôpital Ambroise Paré, Boulogne-Billancourt, France; 6 Department of Medicine (Section of Medical Oncology), Yale School of Medicine, Yale Cancer Center, New Haven, CT USA; 7 Department of Neurology, Yale School of Medicine, New Haven, CT USA

**Keywords:** Lung cancer, Brain metastases, β2M, HLA class-I, IFNγ, APM, CD8 + T-cells

## Abstract

**Background:**

Patients with lung cancer brain metastases can benefit from immune checkpoint inhibitors (ICI). However, intracranial responses are often limited and not always concordant with activity seen in extracranial disease. Defects in IFNγ signaling and HLA class-I antigen presentation machinery (APM) on malignant cells can drive immune evasion and ICI resistance, and have traditionally been viewed as interdependent. The possible role of these alterations in non-small cell lung cancer (NSCLC) brain progression remains poorly understood.

**Methods:**

Using multiplex quantitative immunofluorescence, we measured and spatially mapped IFNγ signaling markers (pSTAT1 and IRF1) and multiple HLA class-I APM components (β2M, PSMB8, PSMB9, PSMB10, TAP1, TAP2, Tapasin, Calreticulin, and ERp57) in cancer cells and neighboring non-malignant stromal cells from two patient cohorts, including primary tumors, intra- and extra-cranial NSCLC metastases. We also studied tumor-infiltrating lymphocyte (TILs) subpopulations in the cohorts, performed whole transcriptomic analysis of parental human NSCLC H2030 cells and their brain metastatic counterpart H2030-BrM3, and expanded the results using spatial transcriptomics of human tumors.

**Results:**

We found comparable levels of IFNγ signaling markers in primary and metastatic lesions and marked downregulation of multiple APM components in metastases, some of which were restricted to the brain. Downregulation of HLA class-I APM components was associated with reduced effector TILs and worse survival. Analysis of human parental H2030 and brain metastatic H2030-BrM3 lung adenocarcinoma cells showed comparable signaling responses after IFNγ stimulation and reduced HLA class-I APM markers in metastatic cells. Transcriptomic analysis of primary/metastatic cells and human tumors identified differential expression of multiple genes associated with HLA class-I APM downregulation.

**Conclusions:**

Our results reveal that APM downregulation is a prominent feature of NSCLC brain metastases, is independent from local IFNγ signaling defects and is associated with unfavorable clinical features. We also identified candidate modulators of the APM pathway in brain metastases with potential translational significance.

**Supplementary Information:**

The online version contains supplementary material available at 10.1186/s12943-026-02687-6.

## Introduction

Up to 40% of patients with non-small cell lung cancer (NSCLC) develop brain metastases (BM), and lung cancer is the most common solid tumor leading to intracranial progression [1]. Patients with NSCLC BM have a poor prognosis with 5-year survival rates of ~ 15%. Despite initial concerns about the potential efficacy of T-cell-based immunostimulatory monoclonal antibodies to access the brain parenchyma and control intracranial disease [2], immune checkpoint inhibitors (ICI) targeting CTLA-4 and the PD-1/PD-L1 axis blockers have shown anti-tumor activity in a subset of patients with NSCLC BM [3–9]. A phase II investigator-initiated clinical trial from our group showed an intracranial overall response rate (icORR) of 29.7% in patients with PD-L1–positive NSCLC and BM [10]. The phase II clinical trial, Atezo-Brain, assessing ICI plus platinum-based chemotherapy in patients with advanced non-squamous NSCLC with untreated BM reported an icORR of 42.7% and an intracranial progression-free survival of 6.9 months [11]. Similar intracranial efficacy (icORR of 41.5%) was recently reported in the phase II NIVIPI-Brain trial using platinum-based chemotherapy and dual ICI with nivolumab and ipilimumab [12]. Despite these encouraging results, most patients with NSCLC BM receiving ICI do not show clinical benefit and the majority of those initially responding develop disease progression within the first year [10–12]. In addition, 13—21% of ICI-treated patients show discordant responses between BM and extracranial lesions [10–12], supporting independent mechanisms of adaptive immune evasion between intra- and extracranial disease [13–16].

To date, little is known about mechanisms mediating primary and acquired resistance to ICI in NSCLC BM. Detailed studies of the brain's tumor microenvironment (TME) are often limited by the low availability of specimens due to the difficulty of obtaining biopsies in this location. A previous study by our group analyzing the TME contexture in primary NSCLC and BM found comparable levels of PD-L1 between intra- and extracranial lesions, but lower levels of TILs and T-cell activation/suppression markers in BM, indicating differences in the modulation of adaptive immune responses between disease locations [16].

Intrinsic mechanisms of ICI resistance in solid tumors include the disruption of IFNγ signaling and loss of HLA class-I antigen presentation machinery (APM) in malignant cells [17]. Deleterious mutations in IFNγ signaling pathway genes encoding JAK1/2 or the HLA class-I APM component Beta-2-microglobulin (β2M) have been reported in NSCLC progressing after initial response to ICI [18–21]. Additional studies support a role of IFNγ signaling defects and loss of HLA class-I APM components in spontaneous adaptive immune evasion as well as in primary ICI resistance [22, 23]. Because IFNγ stimulation can modulate the expression of multiple APM components in both malignant and non-malignant cells, both responses have been traditionally considered to be interdependent. The suppression of IFNγ signaling and APM components dysregulation can occur also as a consequence of epigenetic silencing in the absence of structural genomic alterations, which suggests the possibility of restoring functionality to enhance anti-tumor immunity [22]. The possible contribution, clinical significance and inter-relationship between IFNγ signaling defects and HLA class-I APM alterations in NSCLC BM are currently unknown.

Here we used multiplexed quantitative immunofluorescence (QIF) to simultaneously measure and spatially map IFNγ signaling and the HLA class-I APM in primary lung tumors (PLT), lung brain metastases (LBM) and extracranial metastases (ECM). We also used western blot and whole transcriptome analysis to study paired parental and brain metastatic human NSCLC cells with or without exposure to IFNγ, as well as spatial transcriptomics from human PLT. Our results reveal downregulation of key HLA class-I APM components in brain metastatic NSCLC, independent of IFNγ-signaling defects. In addition, we identify APM abnormalities restricted to LBM and highlight candidate modulators.

## Material and methods

### Patients and samples

Formalin-fixed paraffin-embedded (FFPE) tumor samples from primary and metastatic NSCLC in two retrospective cohorts of patients treated at Yale-New Haven Hospital and represented in tissue microarrays (TMA) were studied (Table 1). The first cohort (Cohort 1) included samples from 87 immunotherapy-naïve patients with 41 PLT, 55 LBM, and 10 ECM collected between 2002 and 2015 [16]. The median age was 64 years (interquartile range 26—83 years) and most patients were female (60%), smokers (80.5%), and with adenocarcinoma histology (64.4%). Half of the patients were diagnosed with stage IV disease (49.5%) and a subset (19.5%) harbored known oncogenic drivers including *KRAS* mutations (*n* = 8), *EGFR* mutations (*n* = 6) and *ALK* rearrangements (*n* = 3). The clinicopathologic characteristics of the subgroup of patients with paired primary/brain metastatic samples were similar to those from the total cohort (supplementary Table S1). Most samples were collected at the time of diagnosis and had not received prior treatment (77%) (supplementary Table S2). The second cohort (Cohort 2) comprised baseline samples from 66 patients treated with ICI between 2018 and 2022, including 47 PLT, 9 LBM, and 10 ECM [24]. As expected for an ICI-treated cohort, Cohort 2 included mostly patients with stage IV disease and smoking history (Table 1). Subsequent treatments received by patients after tissue sampling in cohort 1 and cohort 2 were also collected (supplementary Table S3). An additional cohort (Cohort 3), including 184 patients with primary NSCLC, was analyzed using multi-region spatial transcriptomics and is described in the supplementary Table S4. TMA were constructed as previously reported [25, 26]. Briefly, sections from each tumor block were examined by a pathologist and representative regions including at least 10% of viable cancer cells were selected. The TMA were constructed including one to four 0.6 mm individual cores obtained from different areas of each donor tumor block. The marker scores from all available tumor cores from the same sample were averaged to minimize the possible impact of spatial heterogeneity.

**Table 1:** Clinicopathologic characteristics of Cohorts #1 and #2

Clinical Characteristics	Cohort 1 (*N* = 87)	Cohort 2 (*N* = 66)	*p value*
Gender, n (%)			
Male	35 (40)	32 (48)	*0.5128*
Female	52 (60)	34 (52)	
Age, n (%)			
median (range)	64 (26—83)	66 (37—84)	*0.5157*
≤ 65	47 (54)	32 (48)	
> 65	40 (46)	34 (52)	
Smoking status, n (%)			
Current/Former Smokers	70 (80.5)	63 (95)	*0.0073*
Never Smokers/NA	17 (19.5)	3 (5)	
Stage, n (%)			
I-III	43 (49.5)	14 (21)	*0.0004*
IV	43 (49.5)	52 (79)	
NA	1 (1)	0	
Histology, n (%)			
Adenocarcinoma	56 (64.4)	41 (65)	*0.7385*
Other	31 (35.6)	25 (35)	

### Multiplexed immunofluorescence

Consecutive TMA sections were stained using 5 multiplexed QIF panels, including the markers: 1) 4’−6-diamidino-2-phenylindole (DAPI), pan-cytokeratin (CK), IRF1, pSTAT1 and β2M; 2) DAPI, CK, CD8, CD4 and FOXP3; 3) DAPI, CK, PSMB8, PSMB9 and PSMB10; 4) DAPI, CK, TAP1, TAP2 and CD8; and 5) DAPI, CK, Tapasin, Calreticulin and ERp57. The QIF analysis was performed as previously reported [23, 25, 26]. Briefly, TMA slides were deparaffinized and subjected to antigen retrieval using pH = 8.0 EDTA buffer (Sigma-Aldrich) and boiled for 1 h at 96ºC in a pressure-boiling container (PT module, Lab Vision). Slides were then incubated with methanol and hydrogen peroxide for 30 min at room temperature. Non-specific binding was blocked by a 30-min incubation in 0.3% bovine serum albumin in TBST (TBS with 0.1% Tween 20). Primary and secondary antibodies employed are summarized in the supplementary Table S5. Fluorophore-conjugated reagents were added subsequently after each primary–secondary antibody pair: Cy3-tyramide (1:100, Akoya Biosciences), Cy5-tyramide (1:50, Akoya Biosciences), and biotinylated tyramide (1:50, Akoya Biosciences) followed by streptavidin conjugated with Alexa Fluor 750 (1:100, Thermo Fisher Scientific). Residual horseradish peroxidase activity between incubations with secondary antibodies was eliminated by exposing the slide twice for 7 min to a solution containing benzoic hydrazide and hydrogen peroxide. After each reagent the slides were washed for 30 s with TBS followed by TBST. Finally, the slides were mounted using Prolong Gold. To ensure reproducibility across experiments, an index TMA containing positive and negative control samples previously used for antibody validation and titration was included in each staining run.

### QIF analysis and scoring

Stained slides were scanned using a Vectra Polaris imaging station (PerkinElmer). Quantitative measurement of each fluorescence channel (the QIF score) was calculated by dividing the marker(s) pixel intensity by the area of the selected compartment using the AQUA® software (Navigate Biopharma), allowing objective measurement of markers within user-defined compartments and comparability of scores across different tissue areas and samples [27]. The cancer cell, stromal, and total tissue compartments were defined based on CK positivity (cancer cells), CK negativity (stromal cells), and DAPI positivity (all nucleated cells), respectively. Samples in which the aggregate β2M protein levels were lower in malignant cells than in the adjacent non-tumor stromal cells within the same specimen and showing a mean cancer cell/stromal cell QIF ratio score < 1 were considered as displaying cancer cells β2M downregulation. (E.g.), samples with cancer cell β2M levels comparable to or higher than those in the surrounding non/tumor stromal cells and a mean cancer cell/stromal cell QIF ratio score ≥ 1 were considered as having unaltered β2M expression [23, 28]. All stained slides were examined by a pathologist, and cases with extensive tumor necrosis and/or staining artifacts were excluded from the analysis.

### Cell lines and treatments

For in vitro studies, we used the human lung adenocarcinoma cell line H2030 and its *in vivo-selected* brain metastatic sub-population H2030-BrM3 [29]. The H2030 cell line was established from a lung adenocarcinoma patient and harbors a *KRAS G12C* mutation. The H2030-BrM3 cells were generated through sequential in vivo selection steps, involving repeated rounds of intracardiac injection of parental H2030 cells into immunodeficient mice, followed by isolation and expansion of tumor cells that successfully colonized the brain. These cells can disseminate intracranially after orthotopic implantation in the mouse lung. Both cell lines were cultured under the same conditions with RPMI (11875093, Gibco), 10% Fetal Bovine Serum (12103C, Sigma Aldrich), 5% penicillin–streptomycin (15140122, Gibco) and 0.5% amphotericin B (A2942, Sigma Aldrich) and incubated at 37 °C in a humidified atmosphere containing 5% CO_2_. Cells were passaged every 3–4 days and cultured for at least three days before harvesting. In vitro stimulations were performed by exposure for 24 h with vehicle alone, 20 ng/mL or 100 ng/mL of human recombinant IFNγ (#285-IF, R&D Systems). Cells were periodically authenticated using genomic analysis and tested for Mycoplasma contamination.

### Western Blot analysis

One million H2030 or H2030-BrM3 cells were lysed in RIPA buffer (89900, Thermo Fisher Scientific) and supplemented with a protease and phosphatase inhibitors cocktail (78440, Thermo Fisher Scientific). Protein concentrations were determined using a BCA Protein Assay kit (23227, Thermo Fisher Scientific). Equal amounts of protein (40 µg) were separated by SDS-PAGE using a polyacrylamide gel matrix (4568093, Bio-Rad), transferred to a nitrocellulose membrane, and blocked for 1 h with 5% non-fat milk. Membranes were incubated with the primary antibody overnight at 4ºC. The following primary antibodies were used: Vinculin (rabbit monoclonal antibody, 1:1000, clone E1E9V, Cell Signaling Technology); total STAT1 (rabbit monoclonal antibody, 1:1000, clone D1K9Y, Cell Signaling Technology); phosphorylated STAT1 (rabbit monoclonal IgG, 1:1000, clone 58D6, Cell Signaling Technology); IRF-1 (rabbit monoclonal IgG, 1:1000, clone D5E4, Cell Signaling Technology); PD-L1 (rabbit monoclonal antibody, E1L3N, 1:1000, Cell Signaling Technology); β2-microglobulin (mouse monoclonal IgG2b, 1:1000, clone ab215889, Abcam). After TBS washes, the membranes were incubated with secondary antibodies (HRP-conjugated anti-rabbit/anti-mouse, 1:2000 in 1% non-fat milk) for 1 h at room temperature. The protein detection was performed using enhanced chemiluminescence with SignalFire™ Plus ECL Reagent (#12630, Cell Signaling Technology). Signal quantification was performed using ImageJ 1.53t (National Institutes of Health, USA) and normalized by each corresponding loading control.

### RNA-sequencing data and analysis

Samples containing 1 × 10^6 H2030 or H2030-BrM3 cells treated with vehicle alone or 100 ng/mL IFNγ were collected and kept at −80ºC until use. RNA extraction and whole transcriptome sequencing were conducted as previously reported at the Yale Center for Genome Analysis (YCGA) [28]. The mRNA transcripts were analyzed using TrimGalore (v.0.6.7). Trimmed FASTQ files were evaluated using MultiQC (v.1.10.1). The sequence reads were mapped to the Human genome version Hg38 using HISAT2(v.2.2.1), and SubRead (v.2.0.3) tool was used to quantify the gene expression using feature Count function [30]. Genes read counts were normalized using the edgeR package (v.3.38.4) [31]. DESeq2 R package (v.1.36.0) was employed for differential expression analysis. Differentially expressed genes were selected from the normalized counts using log2 counts per million reads (CPM) between samples and treatment conditions with fold change > 1.5 and false discovery rate < 0.05 [32]. Pathway enrichment analysis was performed using the Enrichr platform, focusing on the Reactome Pathways database [33, 34]. Each gene set was analyzed independently to identify significantly enriched biological pathways. Enrichment significance was determined based on adjusted P-values and combined scores provided by Enrichr. The top-enriched pathways for each gene set were selected based on statistical significance and visualized as bar plots, highlighting the most relevant biological processes associated with the observed transcriptional changes.

### Spatial transcriptomics

Whole transcriptome spatial analysis was performed using the GeoMx® Digital Spatial Profiling platform (NanoString/Bruker, USA). FFPE sections from Cohort 3 were processed following the vendor’s manual slide-preparation protocol (MAN-10150-01), and three TMA blocks containing different areas of the same tumors were analyzed. Sections were deparaffinized, followed by 20 min of heat-mediated antigen retrieval. RNA targets were then exposed with proteinase K for 20 min and slides were hybridized overnight with the human Whole Transcriptome Atlas (WTA) probes. The next day, stringency washes were performed to remove off-target probes and the slides were stained for CK and SYTO 13 for nuclear staining using immunofluorescence protocols. The slides were then washed and loaded onto the DSP-GeoMx instrument. Each TMA spot constituted a region of interest (ROI). Within each ROI, the tissue was segmented into the CK-positive cancer cell compartment, the CK-negative stromal compartment, and the total tissue area using software-defined fluorescence thresholds. Photo-cleaved oligonucleotide tags were released by UV illumination and collected into a 96-well plate. Sequencing libraries were prepared with GeoMx Seq Code primers and unique dual indices (MAN-10117-01). Sequencing was performed by the YCGA using an Illumina NextSeq 2000 instrument. FASTQ files were processed with the GeoMx NGS Pipeline (MAN-10153-01) to generate digital count conversion (.dcc) files for read counts. ROIs were retained only if they met manufacturer-recommended quality controls thresholds. For downstream analysis, raw counts were normalized to CPM. All the spatial transcriptomics analyses were performed specifically in the CK-positive cancer cell compartment.

### Statistical analysis

QIF score comparisons were performed using the Mann–Whitney test for two independent groups, the Wilcoxon signed-rank test for paired samples and Fisher’s exact test for contingency tables. Relationships between continuous scores were analyzed using Pearson correlations, and those between categorical variables were determined using the chi-squared (Χ^2^) test. Survival functions were compared using Kaplan–Meier estimates, and statistical significance was determined using the log-rank test. Overall survival (OS) was calculated from the time of lung cancer or BM diagnosis to death or the last follow-up after 5 years. The OS hazard ratios (HR) were calculated using univariate Cox models. All *p*-values ≤ 0.05 using two-tailed tests were considered as statistically significant. Statistical analyses were performed using GraphPad Prism v.9 and R Studio v.1.3.1093.

## Results

### IFNγ pathway activation and β2M levels in primary and metastatic NSCLC

**Fig. 1:**
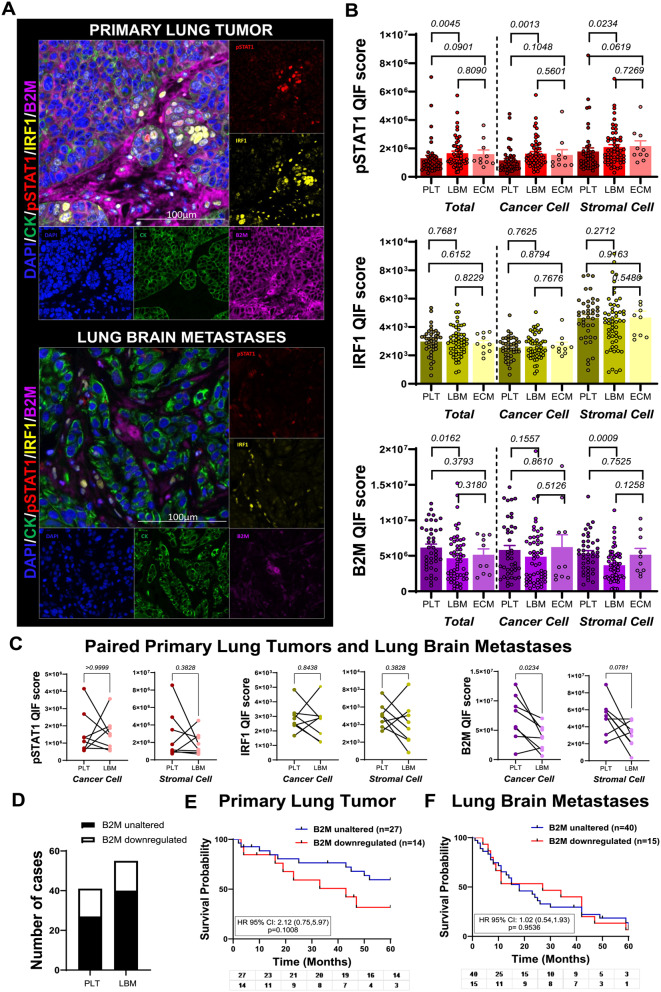
IFNγ signaling and β2M levels in primary and metastatic NSCLC. **A** Representative multi-color immunofluorescence image showing the expression of CK (green), DAPI (blue), pSTAT1 (red), IRF1 (yellow), and β2M protein (purple) in cancer and stromal cells from PLT and LBM. **B** Levels of pSTAT1 (top, red bars), IRF1 (middle, yellow bars), and β2M (bottom, purple bars) measured by QIF in the total tumor tissue area (*Total*), the cancer cell compartment (*Cancer cell*), and the stromal cell compartment (*Stromal cell*) of PLT, LBM, and ECM from Cohort 1. The graphs depict the mean ± SEM of all scores in each group. **C** Graphs showing the levels of pSTAT1, IRF1, and β2M protein measured in the cancer cell and stromal compartment of paired PLT and LBM from the same patients in Cohort 1. **D** Number of cases with unaltered β2M (black bars) and with cancer cell predominant β2M downregulation (white bars) in PLT and LBM. **E**–**F** Kaplan Meier graphical analysis showing the association between cancer cell predominant β2M downregulation and OS in patients with PLT (**E**) and LBM (**F**)

We first studied the localized expression of the IFNγ signaling pathway indicators pSTAT1 and IRF1 and the HLA class-I APM marker β2M protein in the total tumor tissue area, in the CK-positive cancer cell compartment, and in the surrounding non-malignant (CK-negative) stromal cell compartment of samples from Cohort 1. As shown in Fig. 1A, both PLT and LBM showed focal pSTAT1 and IRF1 staining in the nucleus of both malignant and non-malignant cells. As expected, β2M exhibited a cytoplasmic/membranous staining pattern with marked signal reduction in LBM. Quantification of the markers across tumor tissue compartments revealed significantly higher pSTAT1, comparable levels of IRF1, and lower β2M protein in LBM than in PLT (Fig. 1B). Notably, significantly lower β2M expression was seen in the stromal compartment, and a non-significant trend was also observed in the cancer cell compartment of LBM, suggesting a global TME phenomenon. Lower β2M protein levels were also seen in non-tumor brain than in other morphologically normal tissues, indicating lower baseline APM expression in this organ (supplementary Fig. S1). No significant differences in the marker levels were seen between PLT and ECM or between LBM and ECM (Fig. 1B), supporting preservation of IFNγ signaling in both intra- and extra-cranial lesions and selective downregulation of β2M in the brain. Statistically significant lower β2M levels in LBM were also observed in the stromal and cancer cell compartments of the subset of paired PLT and LBM from the same patients included in Cohort 1, supporting the consistency of the findings (Fig. 1C).

A similar number of cases with cancer cell predominant β2M downregulation characterized by lower levels of β2M protein in malignant cells than in neighboring non-malignant cells from the same specimen was observed in PLT and LBM (Fig. 1D). The preferential reduction of β2M expression in malignant cells from PLT was associated with shorter survival (OS HR:2.12; CI:0.75—5.97, Fig. 1E). However, this association was not seen in LBM (HR:1.02; CI:0.54—1.93, Fig. 1F), probably as a consequence of the overall higher risk and lower marker levels in these lesions. We found no consistent association between the presence of cancer cell predominant β2M downregulation and major clinicopathologic variables, including age, gender, smoking history, stage at cancer diagnosis, histology variant, and mutational status (supplementary Table S6). Similar results were observed after stratifying the cohort by median cancer cell β2M levels (supplementary Table S7).

**Fig. 2:**
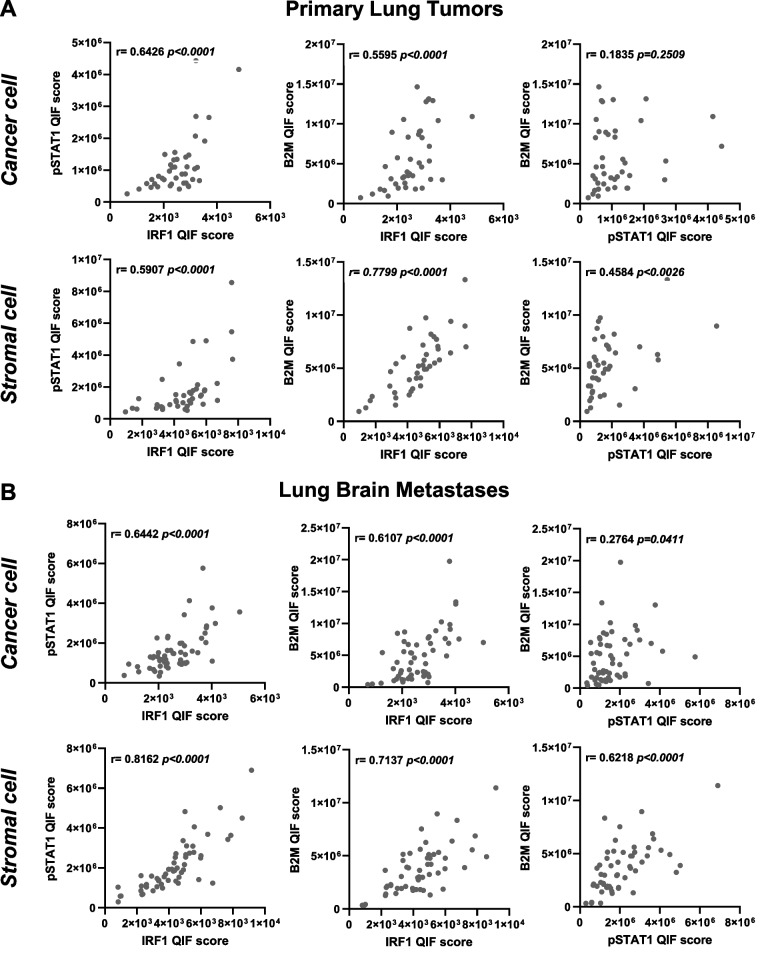
Association between IFNγ signaling and β2M levels in primary NSCLC and brain metastasis. **A**-**B** Graphs showing the correlation between pSTAT1, IRF1, and β2M protein levels in the cancer cell and stromal cell compartments of PLT (**A**) and LBM (**B**) from Cohort 1. Pearson correlation coefficients and p-values are shown within the charts

Because the activation of the IFNγ signaling pathway can modulate the expression of HLA class-I APM components, we examined the association between pSTAT1, IRF1, and β2M levels in the cohort samples. As expected, and shown in Fig. 2, we found a high correlation between the two IFNγ pathway markers across all tumor tissue compartments. The correlation coefficients were similar in PLT (Fig. 2A) and LBM (Fig. 2B), with Pearson’s r values of 0.6426 and 0.6442 in the cancer cell compartment and 0.5907 and 0.8162 in the stromal compartment, respectively. We also found positive correlations between the IFNγ pathway markers and β2M that were numerically higher in LBM than in PLT. Together, these results support the presence of a functional IFNγ signaling pathway in LBM that is independent from, and cannot explain, the observed β2M downregulation in these lesions.

### β2M expression and immune contexture in NSCLC brain metastasis

**Fig. 3:**
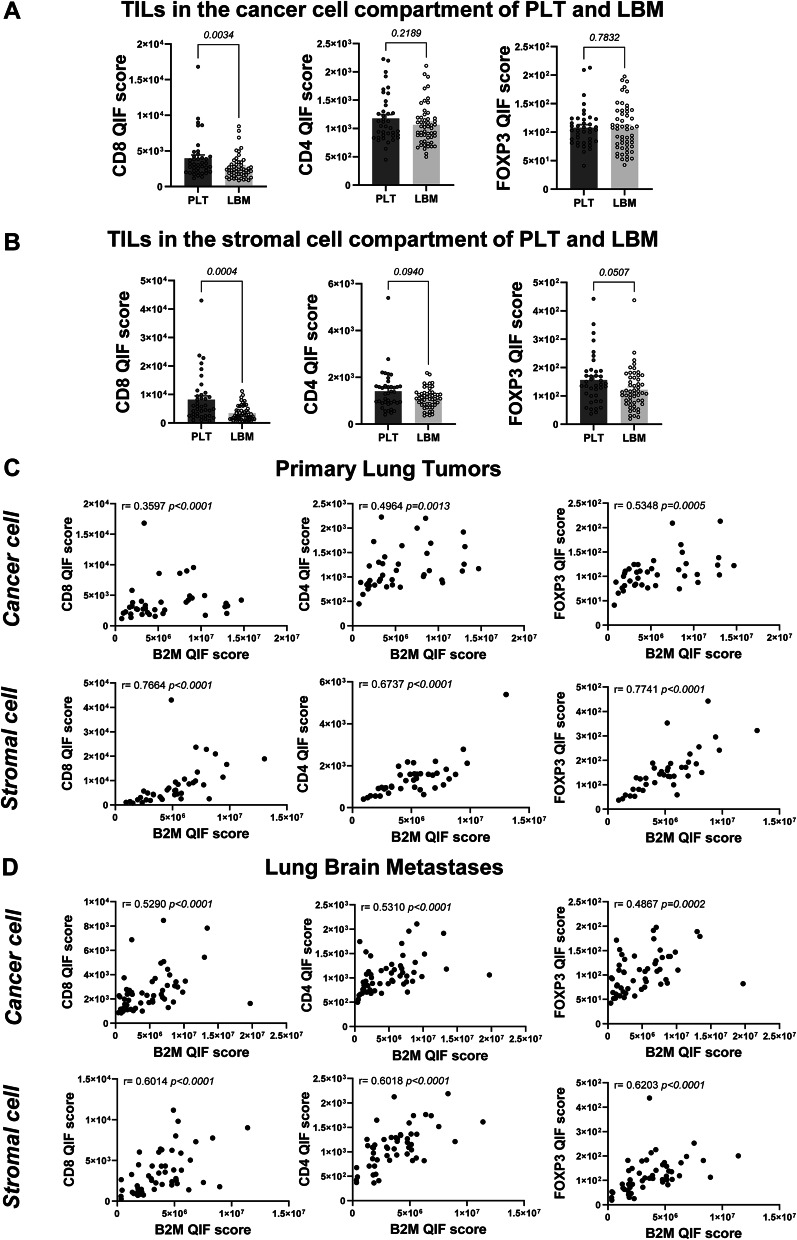
T-cell composition of LBM and association with β2M expression pattern. **A** Levels of CD8 + (left panel), CD4 + (center panel), and FOXP3 + (right panel) TILs measured selectively within the cancer cell compartment by QIF in PLT and LBM from Cohort 1. **B** Levels of CD8 + (left panel), CD4 + (center panel), and FOXP3 + (right panel) TILs measured in the stromal compartment of PLT and LBM. The values reflect the mean ± SEM of all scores. **C**-**D** Graphs showing the correlation between β2M protein and CD8 + (left), CD4 + (center), and FOXP3 + (right) levels in the cancer cell and stromal cell compartments of PLT (**C**) and LBM (**D**) from Cohort 1. Pearson correlation coefficients and p-values are shown within the charts

Given the role of β2M in the presentation of cancer-derived antigens to CD8 + effector T-cells and in mediating malignant cell cytotoxicity, we hypothesized that the β2M levels would be associated with a distinct TILs landscape in LBM. To assess the immune composition of tumor samples, we studied the localized levels of CD8 + effector T-cells, CD4 + helper cells, and FOXP3 + regulatory T-cells (Tregs) using QIF. Consistent with previous observations [16], PLT exhibited higher CD8 + TILs than LBM and similar levels of CD4 + helper T-cells and Tregs in the cancer cell compartment (Fig. 3A). Lower levels of CD8+ TILs population was seen in the stromal compartment of LBM (Fig. 3B). Positive correlations were observed between β2M levels and the different TILs subpopulations (e.g. CD8 +, CD4 +, and FOXP3 +) in the stromal cell compartment of both PLT and LBM. We also found positive correlations between β2M and CD4 + or FOXP3 + cells in the cancer cell compartment of both locations (Fig. 3C-D). LBM with cancer cell predominant β2M downregulation showed a clear trend towards lower CD8 + TILs and similar levels of CD4 + TILs and FOXP3 + Tregs than those without this pattern (supplementary Fig. S2A). As expected, both PLT and LBM with lower levels of pSTAT1, IRF1, and β2M showed fewer TILs than tumors with high levels of the markers (supplementary Figs. S2B-D). Together, these results support that β2M expression has a critical role in modulating local adaptive immune responses in LBM and its downregulation in malignant cells could favor immune evasion.

### IFNγ signaling and β2M expression in an independent NSCLC cohort

**Fig. 4:**
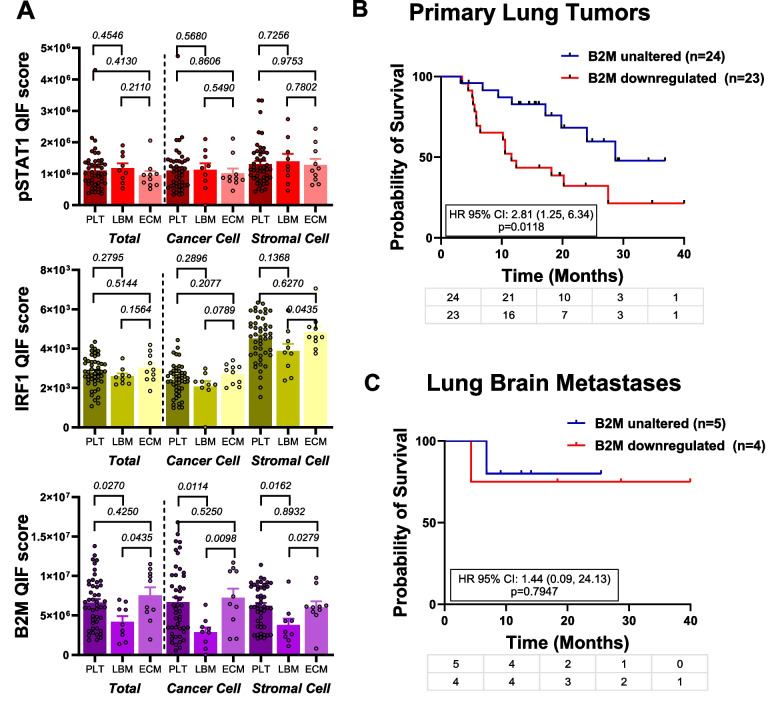
IFNγ signaling and β2M levels in NSCLC from an independent cohort. **A** Levels of pSTAT1 (top, red bars), IRF1 (middle, yellow bars), and β2M (bottom, magenta bars) measured by QIF in the total tumor tissue area (*Total*), the cancer cell compartment (*Cancer cell*), and stromal compartment (*Stromal cell*) of PLT, LBM, and ECM from Cohort 2. The graphs depict the mean ± SEM of all scores in each group. **B**-**C** Kaplan Meier graphical analysis showing the association between predominant cancer cell β2M downregulation and OS in patients with PLT (**B**) and LBM (**C**)

To determine the consistency of our findings, we used QIF to study pSTAT1, IRF1, and β2M protein levels in a second cohort of pre-treatment NSCLC samples from patients receiving ICI (Cohort 2, Table 1). Consistent with the results from Cohort 1, we found similar levels of pSTAT1 and IRF1, but lower expression of β2M protein in the total tissue area, the cancer cell and the stromal compartment of LBM compared to PLT (Fig. 4A). In addition, we found no differences in the expression of these markers between PLT and ECM, and lower levels of β2M in LBM than in ECM, confirming that β2M downregulation is associated with intracranial NSCLC progression. In patients from Cohort 2, cancer cell predominant β2M downregulation in PLT was associated with shorter 5-year OS (HR = 2.81; 95% CI 1.25—6.34) (Fig. 4B), and no survival difference was seen based on the predominant β2M downregulation pattern in the limited set of LBM cases from Cohort 2 (Fig. 4C).

### Expression of additional HLA class-I APM components in primary and metastatic NSCLC

**Fig. 5:**
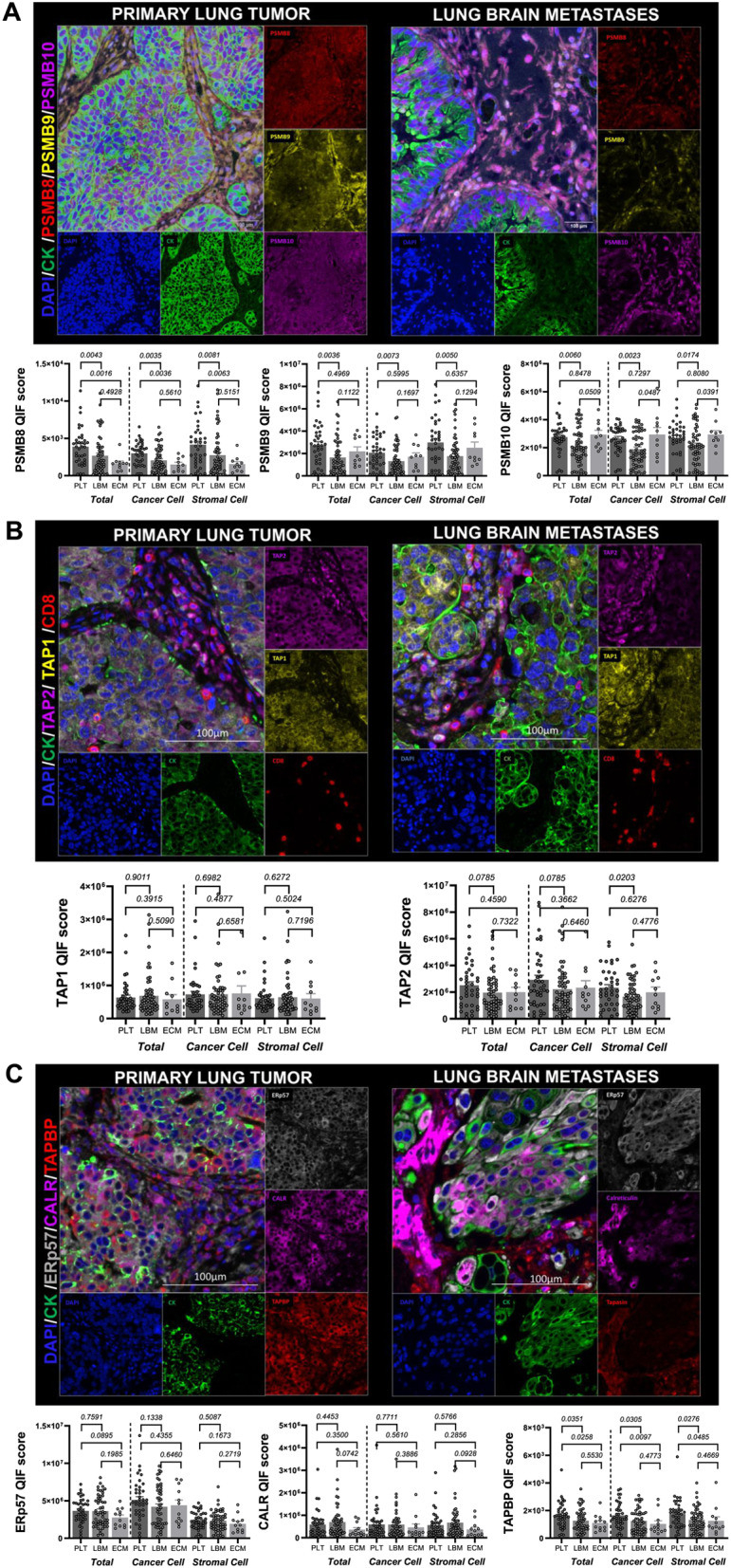
HLA class-I APM components in primary and metastatic NSCLC. **A** Representative multi-color immunofluorescence image showing the localized expression of PSMB8 (red), PSMB9 (yellow), and PSMB10 (purple) in PLT and LBM (top panel). Levels of PSMB 8 (left panel), PSMB9 (middle panel), and PSMB 10 (right panel) in the total tumor tissue area (Total), the cancer cell compartment (Cancer cell), and the stromal compartment (Stromal cell) of PLT, LBM, and ECM. **B** Representative multi-color immunofluorescence image showing the localized expression of TAP1 (yellow), TAP2 (purple), and CD8 (red) (middle panel). Levels of TAP1 (left panel) and TAP2 (right panel) in the total tumor tissue area (*Total*), the cancer cell compartment (*Cancer cell*), and the stromal compartment (*Stromal cell*) of PLT, LBM, and ECM. **C** Representative multi-color immunofluorescence image showing the localized expression of ERp57 (gray), Calreticulin (CALR, purple), and Tapasin (TAPBP, red) (bottom panel). Levels of ERp57 (left panel), Tapasin (TAPBP, middle panel), and Calreticulin (CALR, right panel) in the total tumor tissue area (*Total*), the cancer cell compartment (*Cancer cell*), and the stromal compartment (*Stromal cell*) of PLT, LBM, and ECM. CK (green) and DAPI (blue) were shared across all QIF panels. The graphs depict the mean ± SEM of all scores in each group

The successful generation and intracellular transport of peptides for the production and delivery of surface HLA-peptide complexes requires the sequential involvement of multiple APM components, including the immunoproteasome formed by PSMB8, PSMB9, and PSMB10; the heterodimeric endoplasmic reticulum TAP transporter formed by TAP1 and TAP2; and the protein loading complex formed by Tapasin, Calreticulin, Calnexin, and ERp57 [35–38]. To expand our understanding of HLA class-I pathway alterations in PLT, LBM and ECM, we measured the levels of PSMB8, PSMB9, PSMB10, TAP1, TAP2, ERp57, Calreticulin (CALR), and Tapasin (TAPBP) using QIF panels in cases from Cohort 1. As shown in Fig. 5A-C, all the APM markers showed a cytoplasmic staining pattern with focal expression in both malignant and non-malignant stromal cells. In quantitative analysis, PSMB8 showed lower levels in LBM and ECM than in PLT and this effect was predominant in the cancer cell region (Fig. 5A). PSMB9 and PSMB10 showed lower levels in both cancer cells and in the stromal compartment of LBM than PLT, and no differences were observed between PLT and ECM, suggesting a preferential role in the brain. In addition, PSMB10 showed significantly lower levels in LBM compared with ECM. From the TAP transporter and protein loading complex markers, only TAP2 and Tapasin showed lower expression in LBM than in PLT (Fig. 5B and C). Tapasin also showed lower levels in ECM than in PLT, suggesting a more general role in NSCLC metastasis not limited to intracranial lesions. No significant differences in the levels of TAP1, Calreticulin or ERp57 were observed between different tumor locations. Collectively, these results indicate that multiple HLA class-I components are downregulated during NSCLC progression, with two of them reduced in all metastases regardless of the intra/extra-cranial location (PSMB8 and Tapasin) and three of them downregulated only in intracranial metastasis (β2M, PSMB9, and PSMB10).

### Differential HLA class-I expression and IFNγ responses in parental and brain-metastatic NSCLC cells

**Fig. 6:**
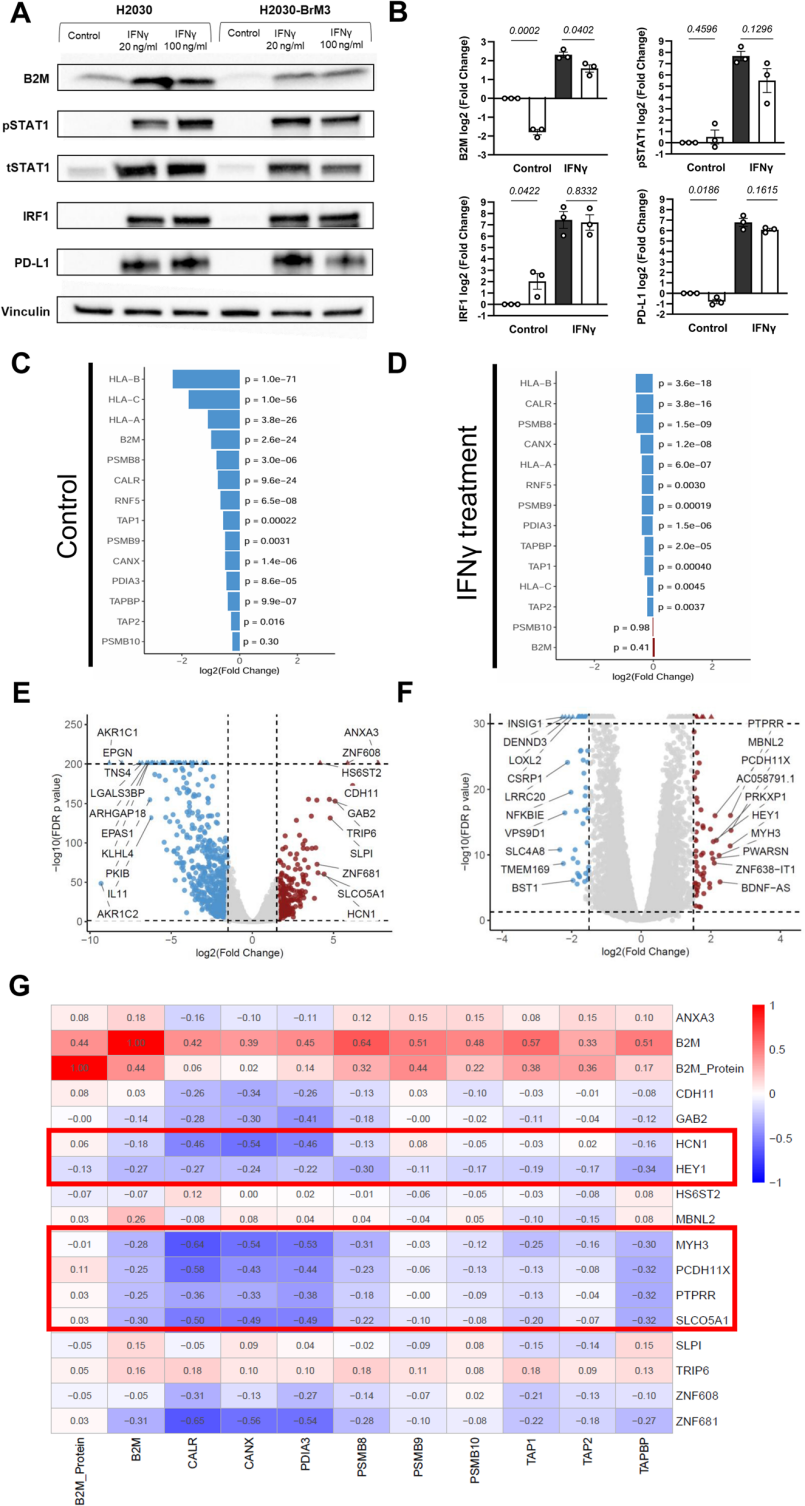
HLA class-I APM expression and IFNγ responses in primary and brain metastatic lung cancer cells. **A** Western blot showing the levels of β2M, phosphorylated STAT1 (pSTAT1), total (tSTAT1), IRF1, and PD-L1 in parental H2030 and H2030-BrM3 BrM3 cells untreated and treated for 24 h with 20 or 100 ng/ml of human recombinant IFNγ. **B** Quantitative densitometric analysis of β2M, pSTAT1, IRF1, and PD-L1 protein levels in H2030 (black bars) and H2030-BrM3 cells (white bars) with or without exposure to 100 ng/ml IFNγ. The results reflect the average of 3 technical replicates, are expressed as log2 fold change relative to control samples, and were normalized by vinculin, used as a protein loading control. **C**-**D** Differential expression analysis of HLA class-I APM genes in H2030-BrM3 cells compared to H2030 without and with exposure to IFNγ. Downregulated genes are shown in blue bars and increased genes in red bars. **E**–**F** Volcano plots showing the top 10 differentially expressed genes in control (**E**) and IFNγ-treated (**F**) H2030 and H2030-BrM3. Genes with > two-fold change and a significant P value after Bonferroni-Holmes correction are highlighted, with those downregulated in H2030-BrM3 shown using blue dots and those upregulated with red dots. **G** Heatmap showing Pearson’s correlation coefficients between the localized expression levels of genes upregulated in H2030-BrM3 cells and HLA class-I APM genes or β2M protein in the cancer cell compartment of primary NSCLC from Cohort 3. Red boxes indicate the genes with the most prominent and consistent negative correlation with HLA class-I transcript levels

For in vitro experiments, we used a previously described model based on the human lung adenocarcinoma cell line H2030 and its brain-metastatic counterpart, H2030-BrM3 [29].To determine the HLA class-I APM state and the ability of parental H2030 and H2030-BrM3 cells to respond to IFNγ stimulation, we measured β2M and JAK/STAT signaling pathway markers (total/phosphorylated STAT1, IRF1, and PD-L1) using western blot in lysates from cultured cell preparations with or without exposure to different concentrations of human recombinant IFNγ. Similar to the results seen in human tumors, H2030-BrM3 cells showed lower β2M protein levels than parental H2030 both at baseline and after IFNγ treatment (Fig. 6A-B). However, pSTAT1, IRF1, and PD-L1 were comparably increased after treatment in both cell lines (Fig. 6A-B), indicating reduced APM expression in the presence of a functional IFNγ signaling pathway. Using transcriptomic analysis, we found that most APM genes exhibited lower expression in the control condition and similar expression in stimulated H2030-BrM3 cells compared with parental cells (Fig. 6C-D). Notably, the *B2M* transcript levels were similar in both cell lines, suggesting a non-transcriptional mechanism involved in β2M protein downregulation in brain metastatic cells. To identify possible negative modulators of HLA class-I APM components in LBM, we performed unsupervised comparative analysis of the transcriptomic data from parental and H2030-BrM3 cells. As shown in Fig. 6E, we identified multiple genes selectively and significantly upregulated in unstimulated brain metastatic cells including *ANXA3, ZNF608, HS6ST2, CDH11, GAB2, TRIP6, SLPI, ZNF681, SLCO5A1* and *HCN1*. From these, *SLPI* encoding the anti-inflammatory protein Secretory Leukocyte Protease Inhibitor, *CDH11* encoding the calcium-dependent intercellular adhesion protein Cadherin-11, and *GAB2* encoding the pleckstrin homology domain adapter protein GRB2-associated-binding protein-2 showed the most prominent and consistent negative correlation with HLA class-I gene expression (supplementary Fig. S3). Genes significantly upregulated in IFNγ-treated brain metastatic cells included *PTPRR, MBNL2, PCDH11X, ACO5879, PRKXP1, HEY1, MYH3, PWARSN, ZNF638-IT1,* and *BDNF-AS* (Fig. 6F)*.* From these, and despite prominent variation across genes, *HEY1* encoding a transcriptional repressor*, MBNL2* encoding an mRNA splicing regulator and *MYH3* encoding the embryonic myosin heavy chain known as myosin-3 showed the most consistent negative correlation with HLA class-I transcripts levels (supplementary Fig. S3). Differential analysis of mRNA pathways showed that the brain metastatic cells display enhanced activation of proliferation-related oncogenic signaling pathways including ERBB4 and PI3K signaling, whereas cell–cell interaction and inflammatory pathways appear reduced (supplementary Fig. S4A). After IFNγ treatment, proliferation-associated pathways remain increased (including MAPK signaling) and cell–cell interaction pathways decreased, whereas the downregulation of inflammatory pathways is no longer apparent (supplementary Fig. S4B).

To further explore the association between the genes upregulated in H2030-BrM3 cells with HLA class-I expression in human malignancies, we conducted multi-region spatial transcriptomics analysis of 184 primary NSCLC (Cohort 3). As shown in Fig. 6G, most of the genes increased in brain metastatic cells are expressed in malignant NSCLC cells and show a negative correlation with cancer cell HLA class-I gene transcripts, with a more prominent effect of *HCN1, HEY1, MYH3, PCDH11X, PTPRR, SLCO5A1, ZNF608 and ZNF681.* Similar trends were observed when comparing the association between *B2M* transcripts or protein levels and expression of other candidate modulatory genes despite prominent methodological differences (Fig. 6G).

## Discussion

Using a multipronged approach including direct quantitative spatial analysis of human tumor cohorts and functional studies in an in vitro NSCLC brain metastases model, our results reveal a prominent role of HLA class-I APM suppression in LBM. The APM downregulation in intracranial lesions occurs in both cancer cells and in the surrounding non-tumor brain parenchyma, shows preferential involvement of specific APM components and occurs in the presence of a functional IFNγ signaling pathway. Our results also show differential transcriptional changes in brain metastatic lung adenocarcinoma cells and identify candidate modulators of HLA class-I APM expression with potential translational implications.

HLA class-I molecules are crucial for cytotoxic T-cell recognition and elimination of cancer, and loss of HLA class-I expression is a well-established mechanism of adaptive immune evasion and ICI resistance. Previously, we reported the non-genomic and cancer cell preferential downregulation of HLA-A, -B, -C, and β2M in 30.4%; and of TAP2 in 44.2% of primary treatment-naïve NSCLC [23, 28]. We also reported cancer cell β2M loss as a mediator of acquired resistance to ICI in patients with advanced NSCLC [20]. Our current results are consistent with these results and support an exacerbated role of APM suppression in LBM characterized by additional reduction in the expression of multiple APM components in brain metastatic NSCLC cells, as well as in the surrounding stroma. This indicates the presence of a permissive TME in LBM with low antigenic potential that is unable to produce strong local adaptive anti-tumor immune responses. This is further supported by a consistent reduction of TILs and T-cell activation markers in LBM relative to primary lesions [16] and reduced TILs subpopulations in LBM with preferential cancer cell β2M downregulation.

Multiple lines of evidence support that the HLA class-I APM is modulated by proinflammatory cytokines such as IFNγ and most tumors lack structural genomic defects in APM genes [23, 28, 39]. Therefore, it has been proposed that the APM reduction or loss in tumors is a dynamic process occurring mainly as a consequence of changes in local cytokine gradients and could be counteracted using immunostimulatory therapies. Our results demonstrate that HLA class-I APM protein downregulation is a consistent feature in LBM and occurs in the absence of detectable alterations in IFNγ signaling. Therefore, APM loss may be a critical, independent, and potentially reversible intrinsic mechanism of adaptive immune evasion and immunotherapy resistance in this setting. In support of this notion, a recent study by our group reported the epigenetic loss of TAP2 as a dominant T-cell evasion mechanism in NSCLC, which was reversible upon treatment with IL-4 receptor blocking antibodies or histone deacetylase inhibitors [28].

Other studies have also reported HLA class-I alterations in NSCLC brain metastasis. Two publications using genomic analysis reported higher HLA class-I gene (e.g. HLA-A, -B, -C, and β2M) loss of heterozygosity in LBM than in PLT [40, 41]. More recently, another study found lower HLA class-I immunostaining in brain metastatic lesions from a cohort of patients with paired PLT and LBM [42]. Our results reveal defects in multiple APM proteins beyond HLA-class I molecules in LBM, including PSMB8, PSMB9, PSMB10, and Tapasin. Moreover, we found preferential downregulation of β2M, PSMB9, and PSMB10 in LBM relative to extracranial lesions, suggesting that the APM loss in the brain is a complex and highly regulated process.

Downregulation of β2M in malignant cells from PLT was associated with worse outcomes in both cohorts. However, a more prominent prognostic impact was observed in Cohort 2. These results could be explained, at least in part, by the larger fraction of cases treated with immunotherapy in this collection. These results are consistent with data from our group and others suggesting a prominent biological role of HLA class-I APM in NSCLC and a prognostic effect of β2M. The limited survival impact of cancer cell β2M downregulation in LBM could be due to the general poor prognosis in these patients and/or the markedly lower expression of this protein in both cancer and surrounding non-tumor cells. The identification of low β2M protein in morphologically normal brain tissue supports limited expression of APM components in this organ. Although previous studies have considered the brain as an “immune sanctuary site” displaying limited adaptive immune responses, clarification of the physiological role, significance, and underlying mechanisms of this finding will require future studies.

Our work identified numerous genes upregulated in brain metastatic lung cancer cells that could be involved in HLA class-I APM modulation. From these, *HEY1* has been nominated as a candidate HLA class-I modulator in gliomas [41] and *CDH11* favors immune evasion in pancreatic ductal adenocarcinoma [42]. *PCDH11X* encodes a protocadherin involved in neural cell–cell interactions and the presence of deleterious somatic mutations in this gene is associated with increased TILs and enhanced benefit to ICI in patients with advanced NSCLC [43]. Additional studies are ongoing to clarify the possible role of *PCDH11X* in APM modulation using in vitro models and genetic perturbations. Other genes, such as *ZNF681, GAB2 *and *MYH3* are also expressed in NSCLC and could exert immunomodulatory and/or oncogenic effects.

Our study has numerous limitations. First, we included retrospective lung cancer cohorts of patients with dissimilar characteristics treated with standard-of-care regimens in which therapies were not standardized or prospectively controlled. In addition, most of the primary/metastatic tumor samples were not from the same patients. Although this could be regarded as a prominent disadvantage, the use of retrospective collections has the potential to more accurately reflect a real-world spectrum of NSCLC cases. In addition, the tumors were represented in TMA format that could over- or under-represent the marker levels. However, the consistent results obtained across independent cohorts support the reliability of the findings. In addition, each tumor sample included in the TMA had at least 2 cores from different tumor regions to minimize the possible negative impact of intratumor spatial heterogeneity.

Due to the restricted availability of tumor tissue samples, we included a limited number of LBM and could not perform comprehensive genomic analysis to establish the oncogenic driver context and presence of deleterious mutations in HLA class-I APM genes or other structural alterations in the cohorts. In addition, the spatial transcriptomic analysis was performed in a PLT cohort limiting its ability to directly address the features associated with β2M loss in LBM.

Finally, the H2030 cell line model used in our experiments may not represent all NSCLC subsets and additional studies will be required to generalize the findings including the use of additional model systems and functional analyses. Additional work will also be required to unravel the mechanisms mediating APM modulation in the context of NSCLC LBM.

Collectively, our results support a prominent role of HLA class-I APM suppression in adaptive immune evasion and intracranial NSCLC progression. The reduction of APM expression in this setting is independent of IFNγ signaling defects, shows specific protein expression patterns, occurs in distinct transcriptomics context and has translational potential for the development of biomarkers and/or novel immunotherapies.

## Supplementary Information

Supplementary Material 1: Supplementary Figure S1. B2M protein levels across normal tisues. A) QIF scores of B2M in the total tissue are of a lymph node, tonsil, kidney, placenta, and brain tissue, respectively. The graphs depict the mean +- SEM of all scores in each group. B) Representative immunofluorescence image illustrating B2M high protein expression in a lymph node (A) and B2M negative expression in a normal brain tissue (right). Supplementary Figure S2. A) Levels of CD8+ (left), CD4+ (center), and FOXP3+ TILs (right panel) in samples of LBM from Cohort 1 classified by cancer cell β2M unaltered (unalt) versus downregulated (down). B-C) QIF scores of CD8+ (left), CD4+ (center), and FOXP3+ TILs (right panel) measured selectively within the cancer cell compartment (top) and stromal cell compartment (bottom) in samples classified as High (H) vs Low (L) (using the media as the cut point) for B2M (B), pSTAT1 (C) and IRF1 (D) QIF in PLT (black dots) and LBM (white dots) from Cohort 1. The graphs depict the mean ± SEM of all scores in each group. Supplementary Figure S3. Heatmap showing Pearson’s correlation coefficients between the expression of genes upregulated in control (A) and IFNγ treated H2030-BrM3 cells (B) and HLA class-I APM genes. Supplementary Figure S4. Signaling pathways upregulated (green bars) and downregulated (blue bars) in the H2030-BrM3 cell line compared to the parental H2030 cell line in control/unstimulated condition (A) and after INFγ treatment (B).

Supplementary Material 2. Original western blot files including all the proteins evaluated

## Data Availability

Raw data included in the manuscript can be requested by email to Dr. Kurt A. Schalper.

## Abbreviations

HLA: Human leucocyte antigen
IFNγ: Interferon gamma
ICI: Immune checkpoint inhibitors
APM: Antigen presentation machinery
NSCLC: Non-small cell lung cancer
TILs: Tumor-infiltrating lymphocytes
BM: Brain metastases
IcORR: Intracranial overall response rate
TME: Tumor microenvironment
PD-L1: Programmed death-ligand 1
β2M: Beta-2-microglobulin
QIF: Quantitative immunofluorescence
PLT: Primary lung tumors
LBM: Lung brain metastases
TMA: Tissue microarrays
ECM: Extracranial metastases
DAPI: 4’-6-Diamidino-2-phenylindole
CK: Cytokeratin
IRF1: Interferon regulatory factor 1
pSTAT1: Phosphorylated signal transducer and activator of transcription 1
CD8: Cluster of differentiation 8
CD4: Cluster of differentiation 4
FOXP3: Forkhead box P3
PSMB8: Proteasome subunit beta type-8
PSMB9: Proteasome subunit beta type-9
PSMB10: Proteasome subunit beta type-10
TAP1: Transporter associated with antigen processing 1
TAP2: Transporter associated with antigen processing 2
TAPBP: Tapasin
CALR: Calreticulin
ERp57: Endoplasmic reticulum resident protein 57
OS: Overall survival
HR: Hazard ratios
Tregs: FOXP3 + regulatory T-cells
NA: Not available
FFPE: Formalin-fixed paraffin-embedded
