# Supplementary material for: NSCLC brain metastases exhibit reduced HLA-I antigen presentation machinery and immune evasion independent of IFNγ signaling defects
Source: Mol Cancer. 2026 May 28;25:197. doi: 10.1186/s12943-026-02687-6 (PMC13471625; doi:10.1186/s12943-026-02687-6)
Supplement: Supplementary file 1 [file 12943_2026_2687_MOESM1_ESM.pdf]

Supplementary Figure S1: Levels of β2M protein across morphologically normal human tissues

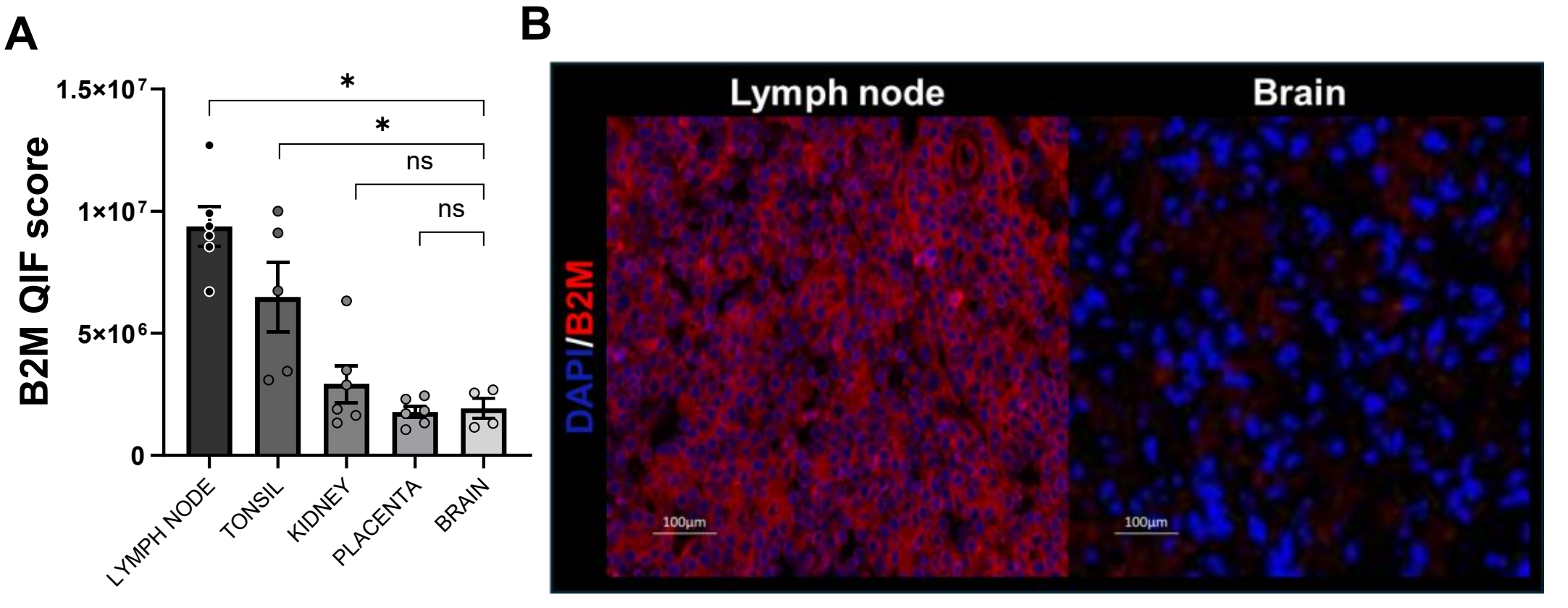

Supplementary Figure S2: A) TILs infiltration in LBM from Cohort #1 in samples classified as B2M unaltered/downregulated. B-C) TILs infiltration in PLT and LBM from Cohort #1 in samples classified as high vs low for B2M, pStat1 and IRF1

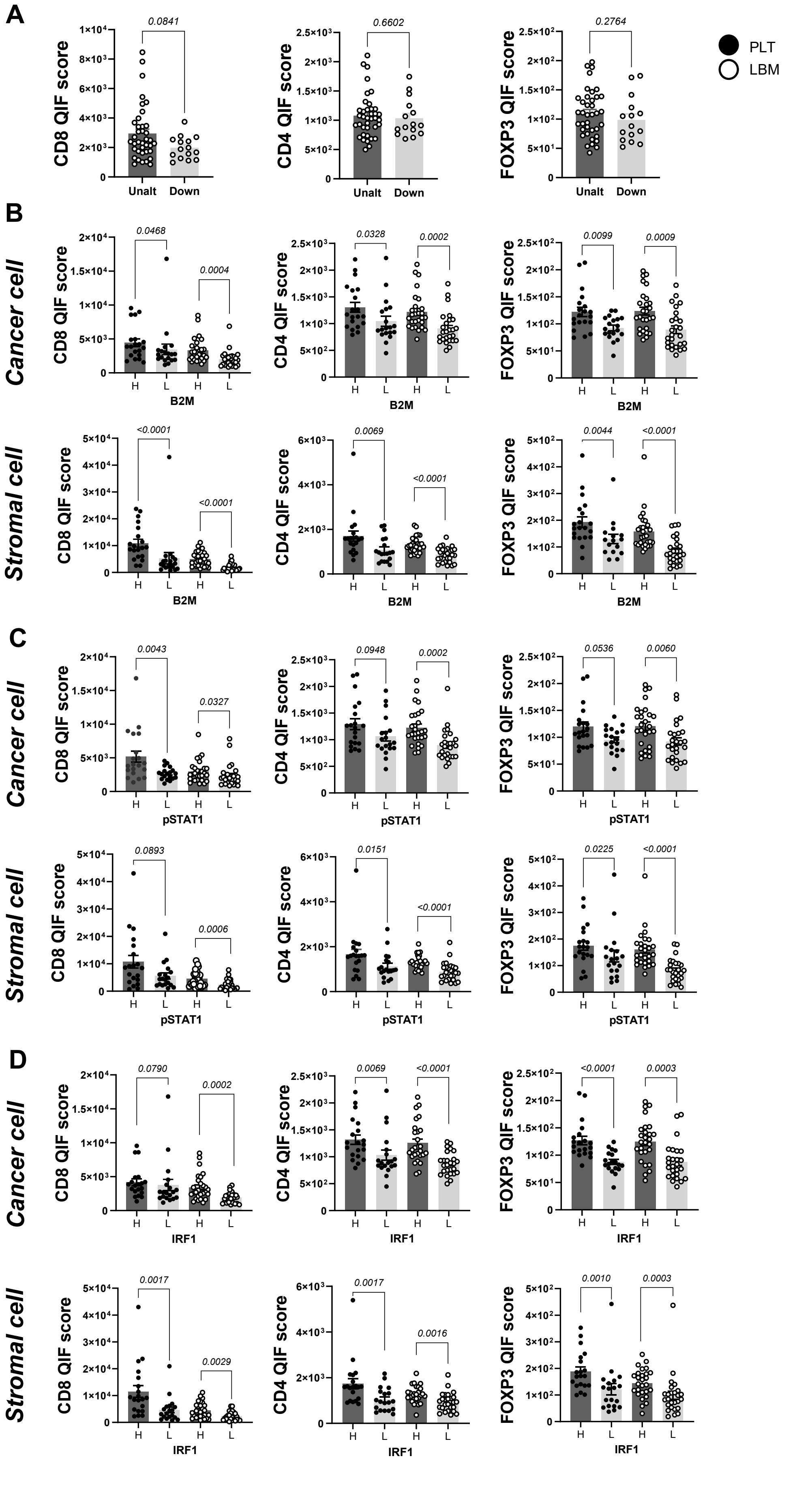

Supplementary Figure S3. Correlation between the expression of genes upregulated in control (A) and IFNγ-treated H2030-BrM3 cells (B) and HLA class-I APM genes

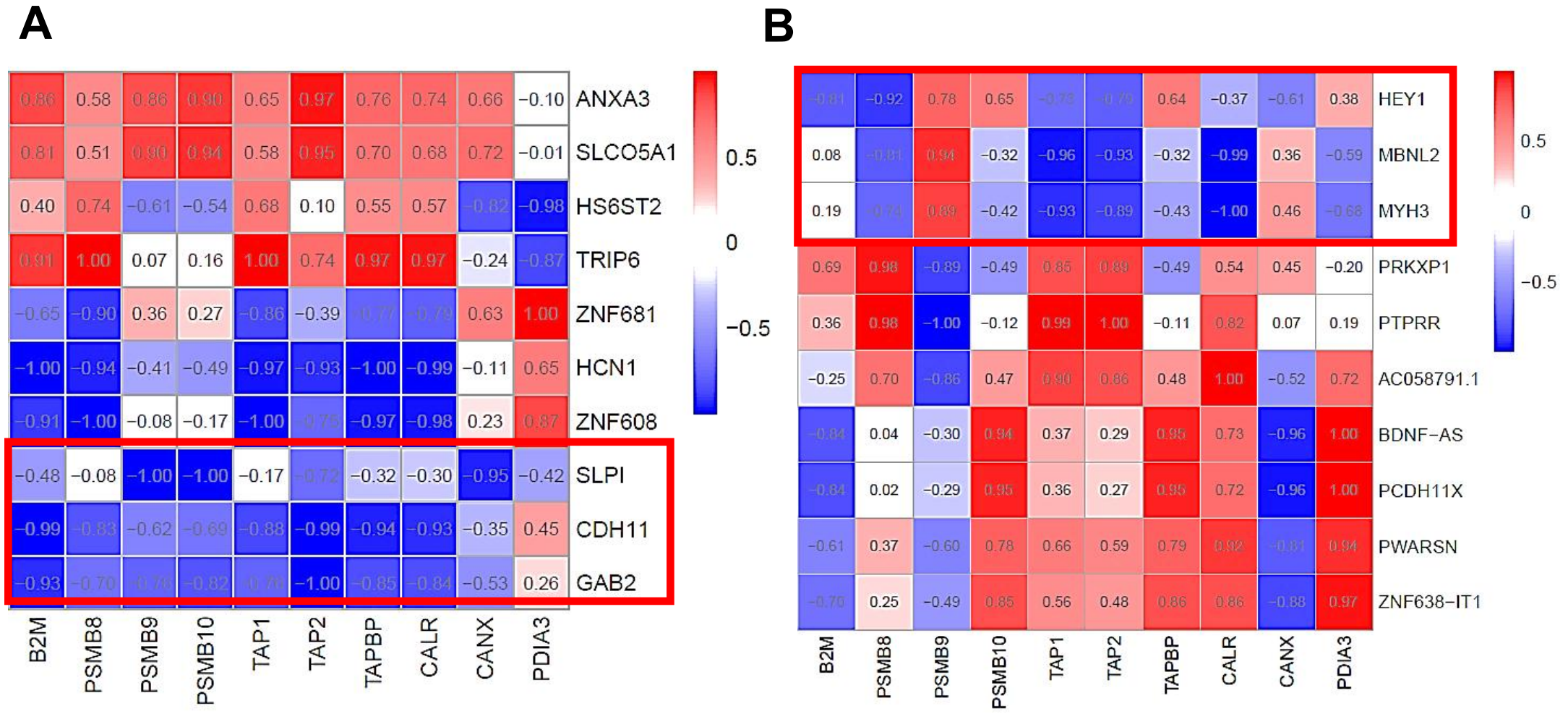

Supplementary Figure S4. Pathway analysis of transcriptomic profile of H2030 and H2030-BrM3 cells

A

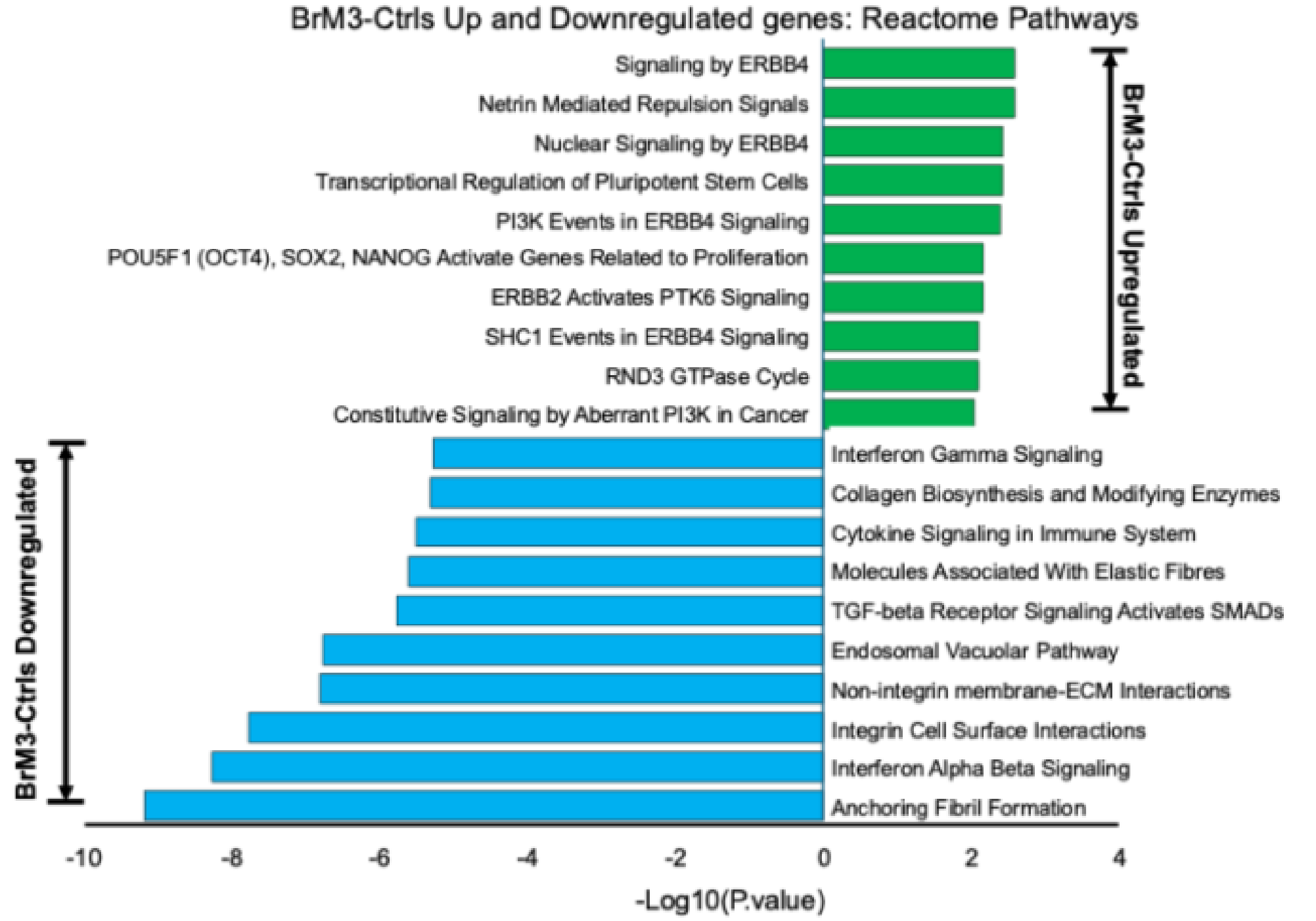

B

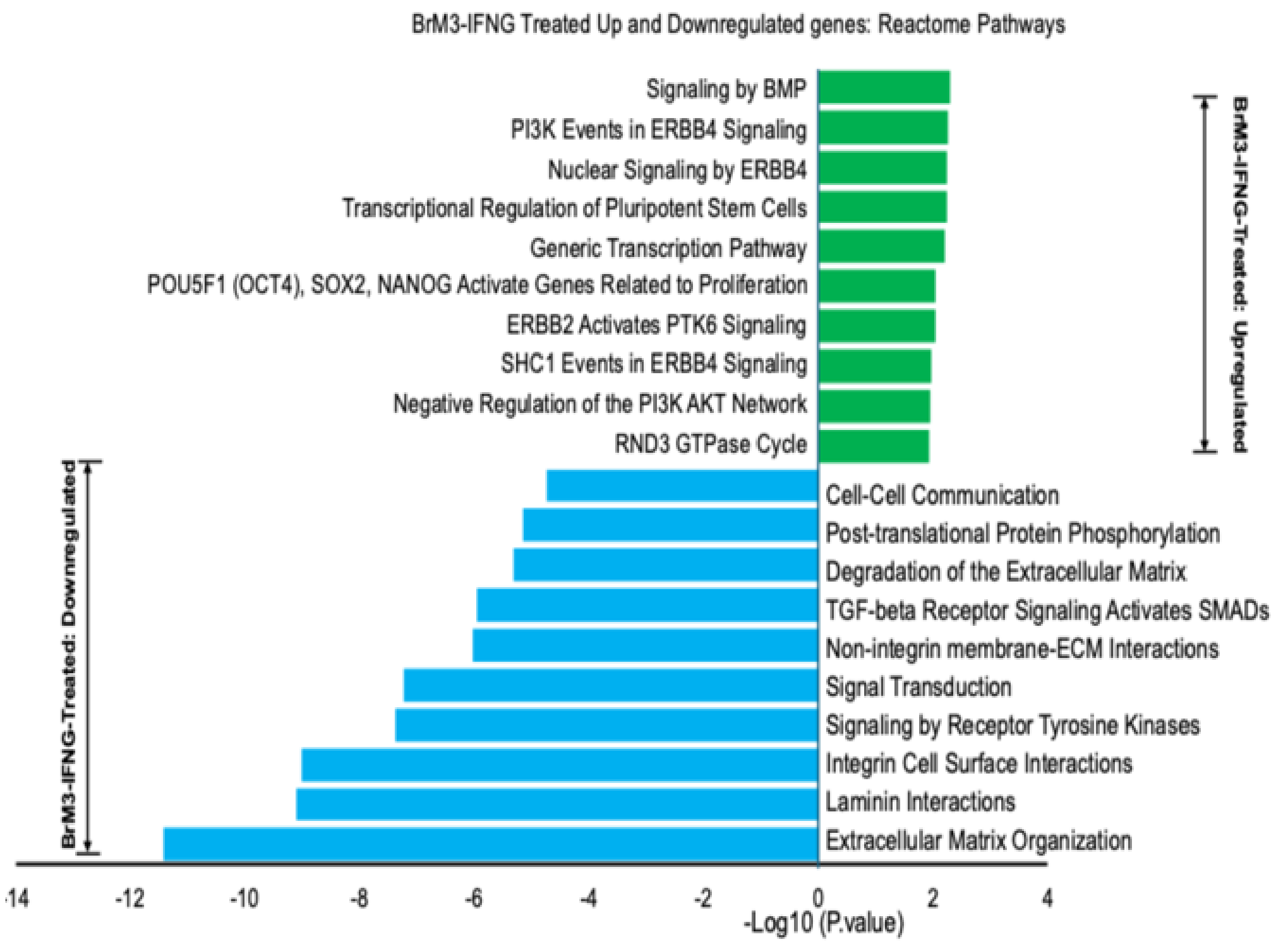

Supplementary Table S1: Clinicopathological characteristics between Cohort #1 and the patients with paired samples

Clinical Characteristics	All patients (N=87)	Patients with paired samples (N=8)	*p value*
**Gender, n (%)**			
Male	35 (40)	3 (37.5)	0.9999
Female	52 (60)	5 (62.5)	
**Age, n (%)**			
median (range)	64 (26-83)	60 (29-76)	
≤ 65	47 (54)	5 (62.5)	0.7251
> 65	40 (46)	3 (37.5)	
**Smoking status, n (%)**			
Current/Former Smokers	70 (80.5)	7 (87.5)	0.9999
Never Smokers/NA	17 (19.5)	1 (12.5)	
**Stage at tumor diagnosis, n (%)**			
I-III	43 (49.5)	5 (62.5)	0.7148
IV	43 (49.5)	3 (37.5)	
NA	1 (1)		
**Histology, n (%)**			
Adenocarcinoma	56 (64)	7 (87.5)	0.2597
Other	31 (36)	1 (12.5)	
**Molecular alterations, n (%)**			
EGFR/KRAS/ALK	17 (19.5)	3 (37.5)	0.3587
Unknown/NA	70 (80.5)	5 (62.5)	

Supplementary Table S2: Treatments prior tissue sampling in Cohort #1

Cohort 1	N=87
**None, n (%)**	67 (77)
**Type of prior treatment, n (%)**	20 (23)
Chemotherapy/Targeted therapy	8 (9)
Prior radiation	6 (7)
Chemotherapy plus radiotherapy	5 (6)
Chemotherapy plus immunotherapy	1 (1)

Supplementary Table S3: Systemic treatments after tissue sampling in Cohort #1 and Cohort #2.

**Cohort 1**	**N=87**
**None, n (%)**	35 (40.2)
**Type of treatment, n (%)**	52 (59.8)
Chemotherapy plus targeted therapy	1 (1.9)
Chemotherapy plus ICI	1 (1.9)
Chemotherapy	33 (63.5)
Targeted Therapy	9 (17.3)
Unknown	8 (15.4)
**Cohort 2**	**N=66**
**ICI, n (%)**	66 (100)
**Type of ICI treatment, n (%)**	
Anti PD-1	49 (74)
Anti-PD-L1	15 (23)
Combo with anti-CTLA4	2 (3)

Supplementary Table S4. Clinicopathologic characteristics of Cohort #3.

Clinical Characteristics	All patients (N=184)
**Gender, n (%)**	
Male	87 (47)
Female	97 (53)
**Age, n (%)**	
≤ 70	106 (58)
> 70	78 (42)
**Smoking status, n (%)**	
Current/Former Smokers	160 (87)
Never Smokers/NA	24 (13)
**Stage at tumor diagnosis, n (%)**	
I-II	165 (90)
III-IV	18 (10)
**Histology, n (%)**	
Adenocarcinoma	144 (78)
Other	40 (22)

Supplementary Table S5: Summary of primary and secondary antibodies employed in QIF

Antibodies	Source	Clone
Cytokeratin Alexa-488 (mouse monoclonal IgG1, 1:100)	eBioscience	AE1/AE3
β2-microglobulin (mouse monoclonal IgG2b, 1:1000)	Abcam	AB215889
Tapasin (rabbit monoclonal IgG, 1:10000)	Cell Signaling Technology	E6P2Z
Calreticulin (mouse monoclonal IgG1, 1:1000)	Abcam	FMC75
ERp57 (rabbit monoclonal IgG, 1:500)	Abcam	EPR10678(B)
TAP1 (mouse monoclonal IgG1k, 1:100)	NA	NOB1 [26]
TAP 2 (mouse monoclonal IgG1k, 1:250)	NA	NOB2 [26]
phosphorylated STAT1 (rabbit monoclonal IgG, 1:200)	Cell Signaling Technology	58D6
IRF-1 (rabbit monoclonal IgG, 1:500)	Cell Signaling Technology	D5E4
CD8 (rabbit monoclonal IgG, 1:250)	Cell Signaling Technology	D8A8Y
CD4 (rabbit monoclonal IgG, 1:100)	Spring Bioscience	SP35
FOXP3 (rabbit monoclonal IgG, 1:25)	Cell Signaling Technology	D2W8E
PSMB8 (mouse monoclonal, IgG1, 1:5000)	Cell Signaling Technology	1A5
PSMB9 (rabbit monoclonal, IgG, 1:2000)	Cell Signaling Technology	E7J1L
PSMB10 (rabbit monoclonal, IgG, 1:2000)	Cell Signaling Technology	E6R7O
PowerVision HRP anti-mouse IgG	Leica Biosystems	PV6114
PowerVision Poly-HRP anti-Rabbit IgG	Leica Biosystems	PV6119
anti-rabbit Envision	Dako	K4002
anti-mouse Envision	Dako	K4001
Rat anti-Mouse IgG1 conjugated with HRP	eBioscience	M1-14D12
Goat polyclonal anti-rabbit IgG conjugated with HRP,1:200	Abcam	AB6721

Supplementary Table S6. Association between β2M cancer cell downregulation and clinicopathologic characteristics in PLT and LBM from patients in Cohort #1

Clinical Characteristics	PLT			LBM		
	Un-altered	Down-regulated	*p value*	Un-altered	Down-regulated	*p value*
**Gender, n**						
Male	13	7	0.99	12	6	0.53
Female	14	7		28	9	
**Age, n**						
≤ 65	13	6	0.99	23	11	0.35
> 65	14	8		17	4	
**Smoking status, n**						
Current/Former Smokers	21	1	0.99	34	12	0.69
Never Smokers/NA	6	3		6	3	
**Stage at tumor diagnosis, n**						
I-III	22	11	0.99	10	6	0.33
IV	5	3		30	9	
**Histology, n**						
Adenocarcinoma	16	9	099	29	11	0.99
Other	11	5		11	4	
**Molecular alterations, n**						
EGFR/KRAS/ALK	3	1	0.99	11	6	0.51
Unknown/NA	24	13		29	9	

Supplementary Table S7. Association between the median β2M protein levels and clinicopathologic characteristics in PLT and LBM from Cohort #1

Clinical Characteristics	PLT		*p* value	LBM		*p* value
	High	Low		High	Low	
**Gender, n**						
Male	10	10	0.99	10	8	0.57
Female	10	11		17	20	
**Age, n**						
≤ 65	11	8	0.35	16	18	0.78
> 65	9	13		11	10	
**Smoking status, n**						
Current/Former Smokers	19	13	0.02	22	24	0.73
Never Smokers/NA	1	8		5	4	
**Stage at tumor diagnosis, n**						
I-III	16	17	0.99	7	9	0.77
IV	4	4		20	19	
**Histology, n**						
Adenocarcinoma	11	14	0.53	19	21	0.77
Other	9	7		8	7	
**Molecular alterations, n**						
EGFR/KRAS/ALK	2	2	0.99	10	12	0.78
Unknown/NA	18	19		17	16	
