# Supplementary material for: NSCLC brain metastases exhibit reduced HLA-I antigen presentation machinery and immune evasion independent of IFNγ signaling defects
Source: Mol Cancer. 2026 May 28;25:197. doi: 10.1186/s12943-026-02687-6 (PMC13471625; doi:10.1186/s12943-026-02687-6)

B-2 microglobulin

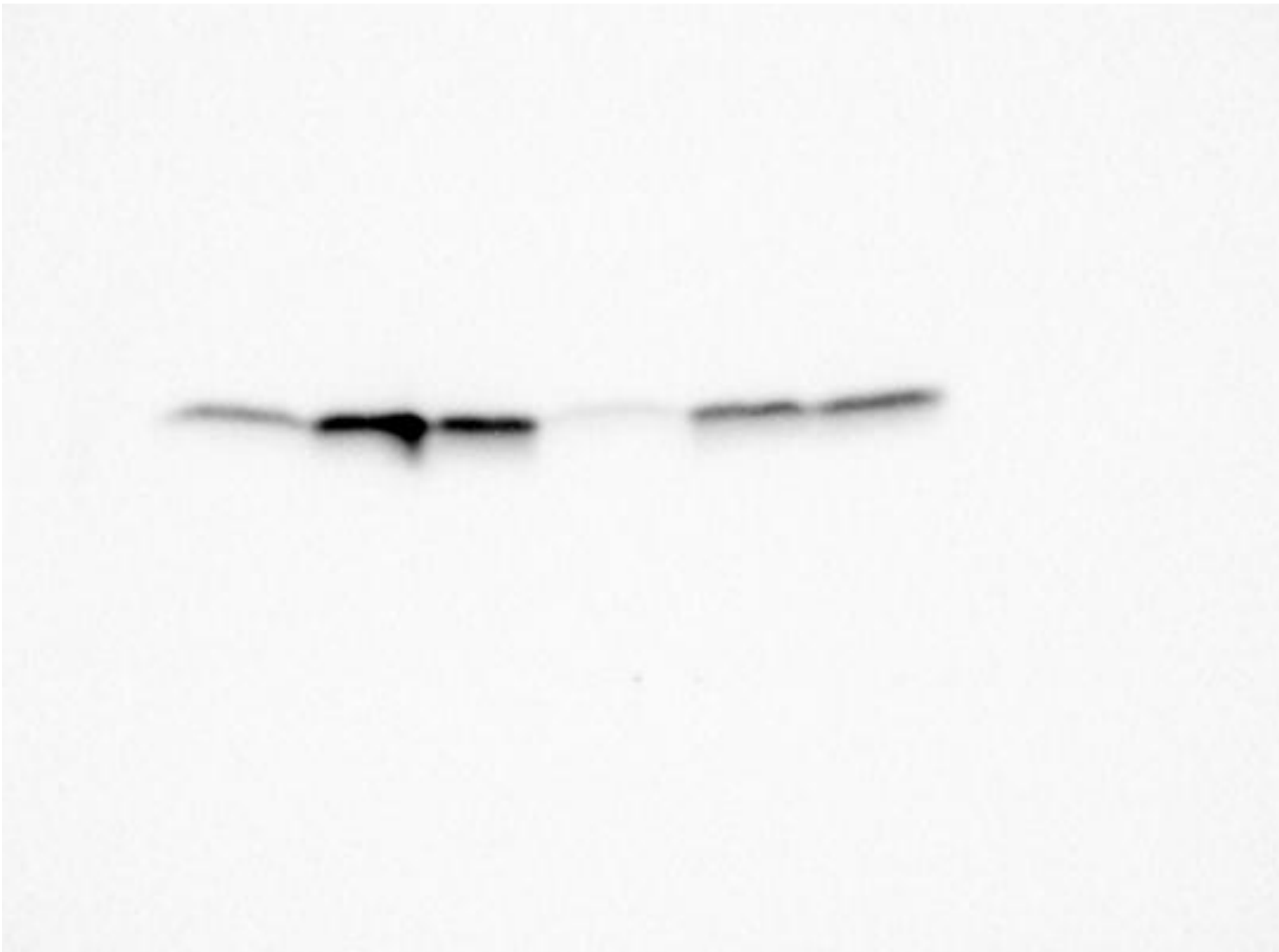

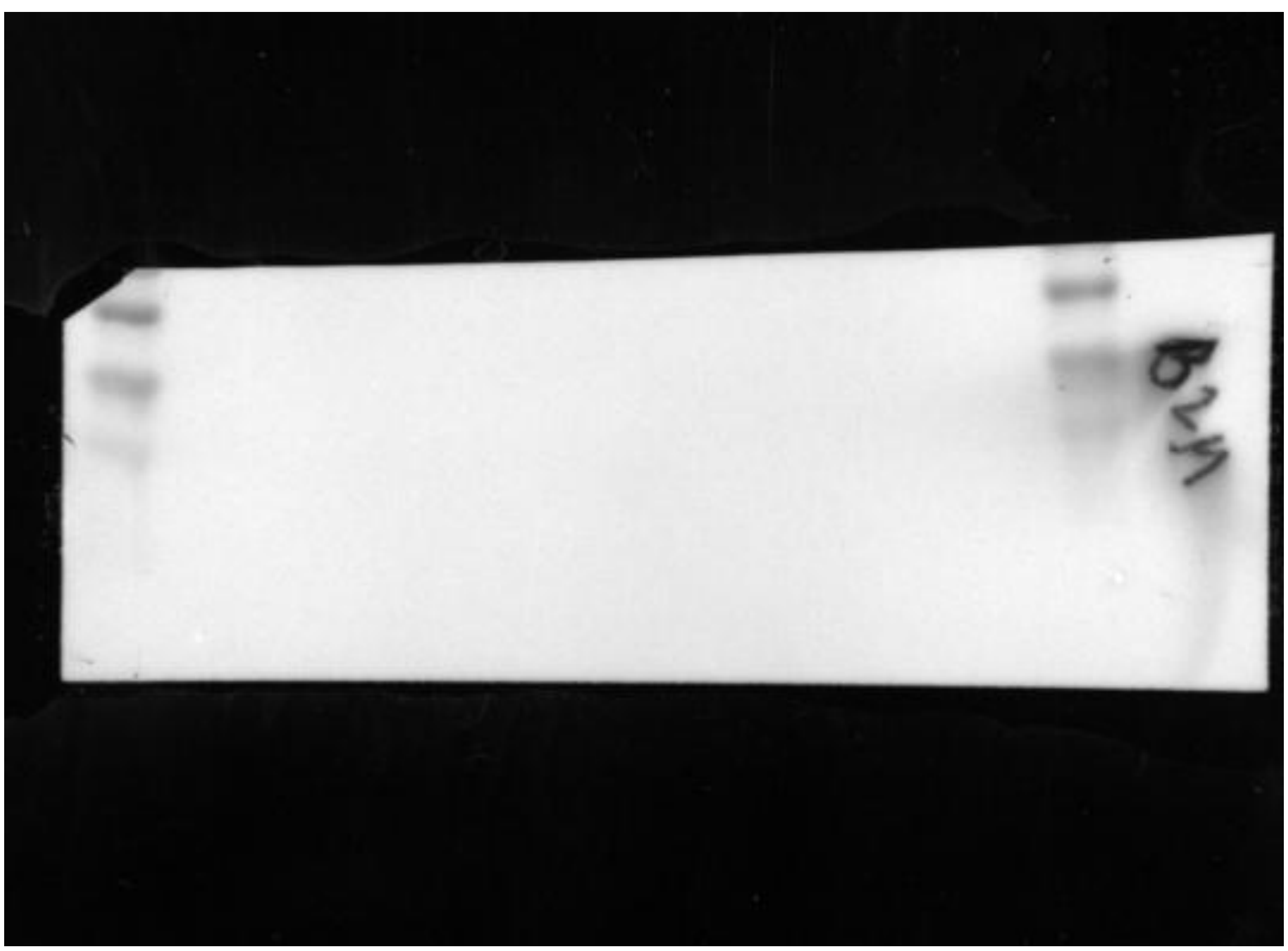

IRF1

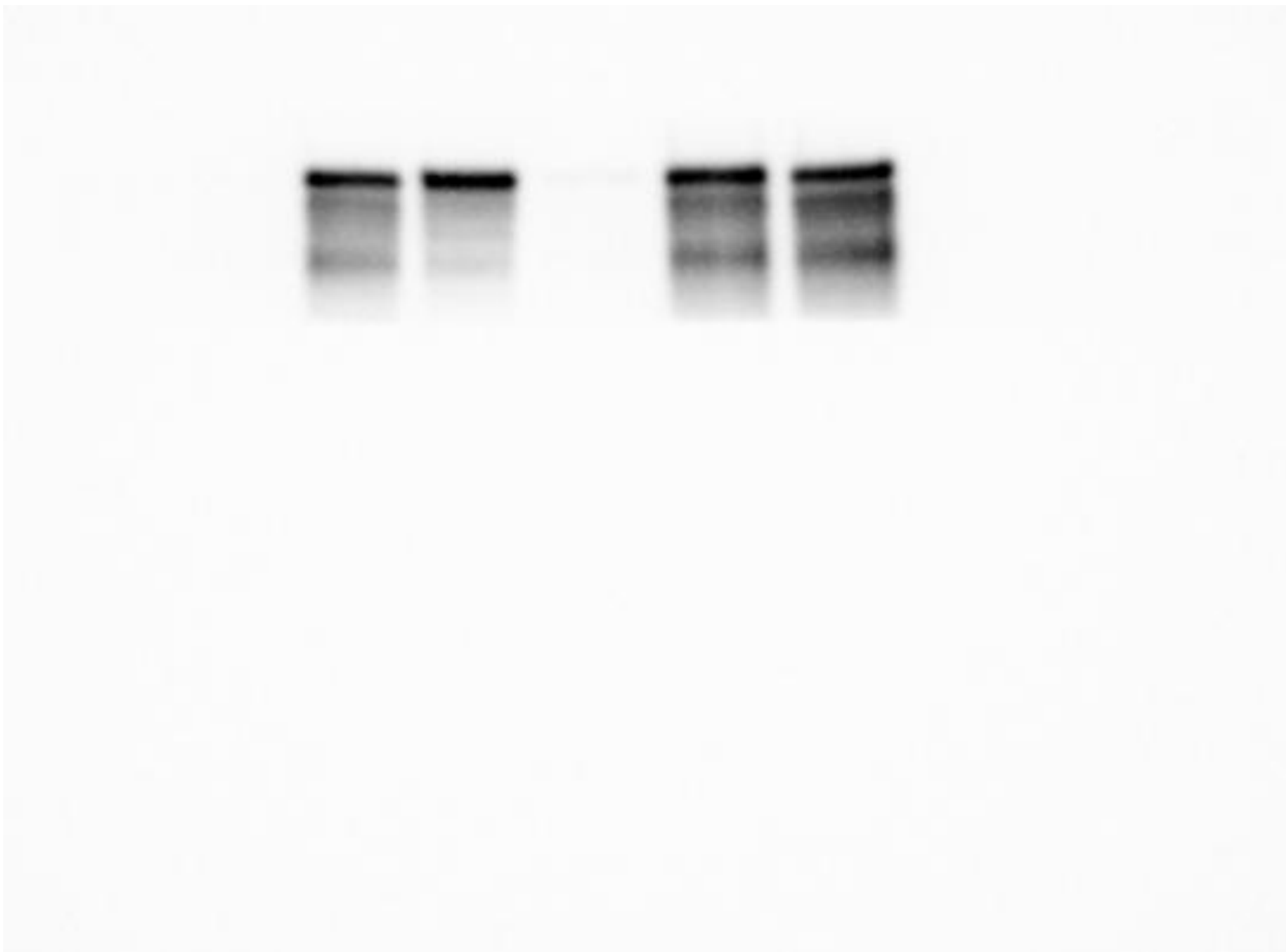

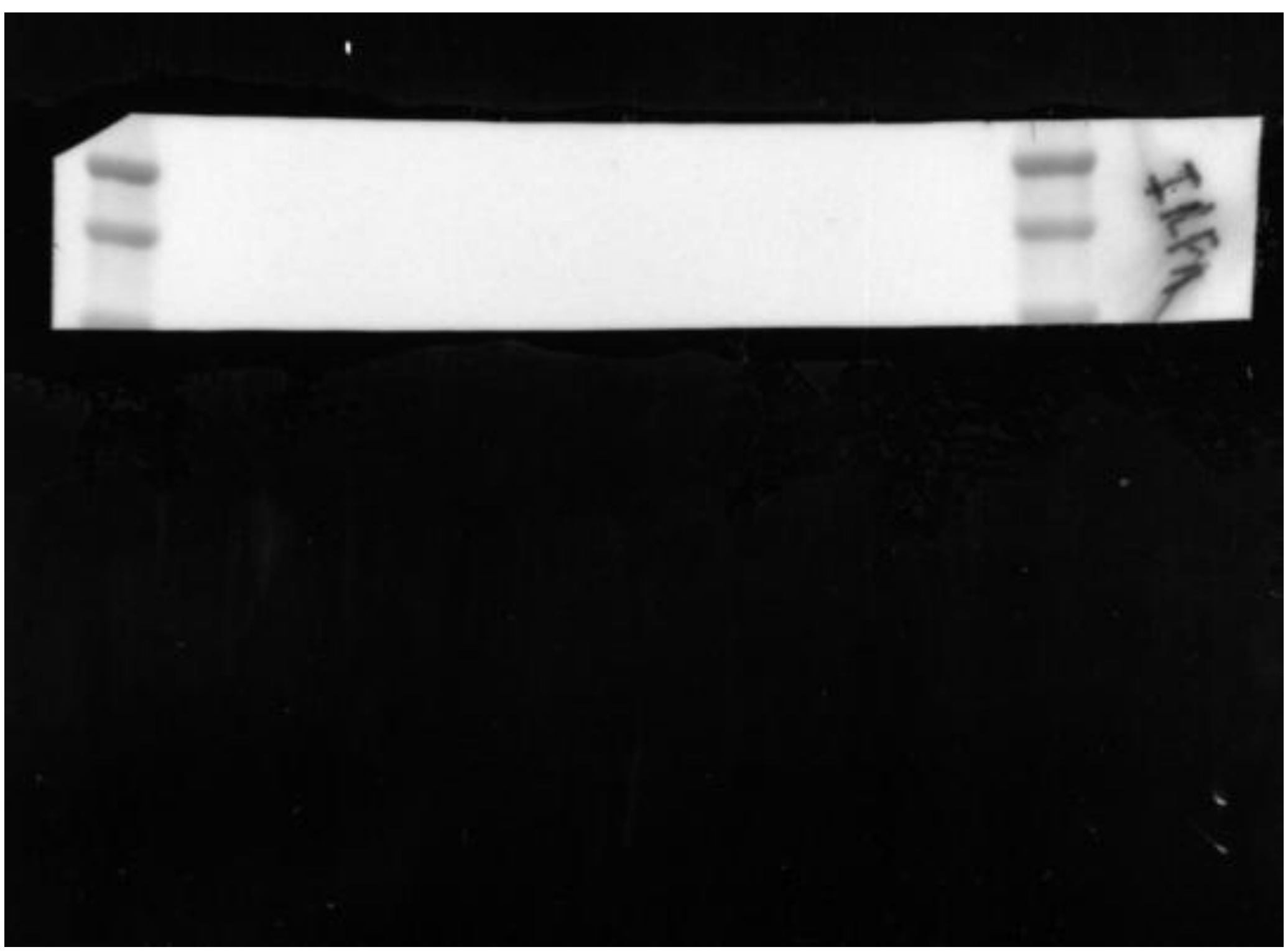

PD-L1

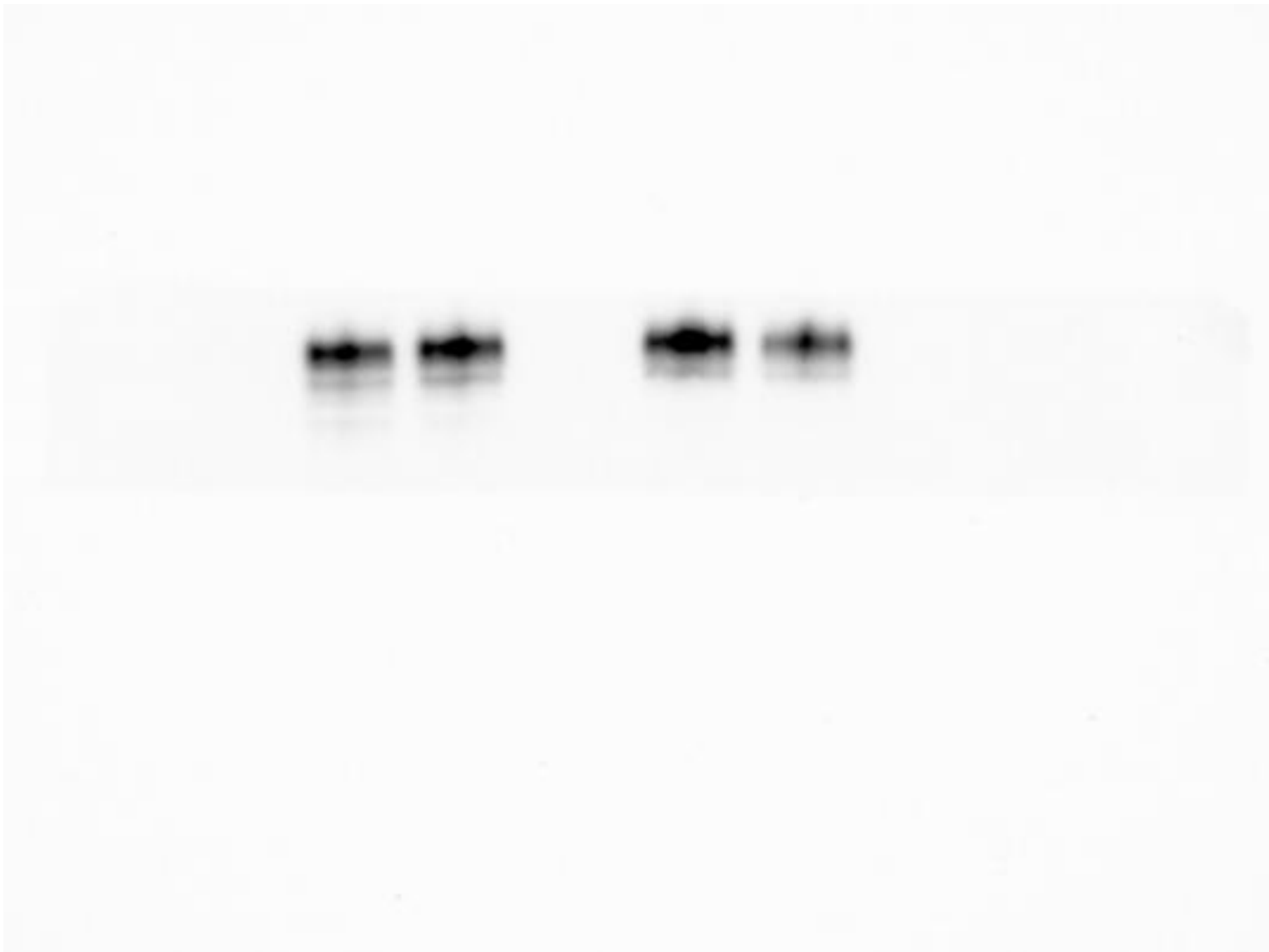

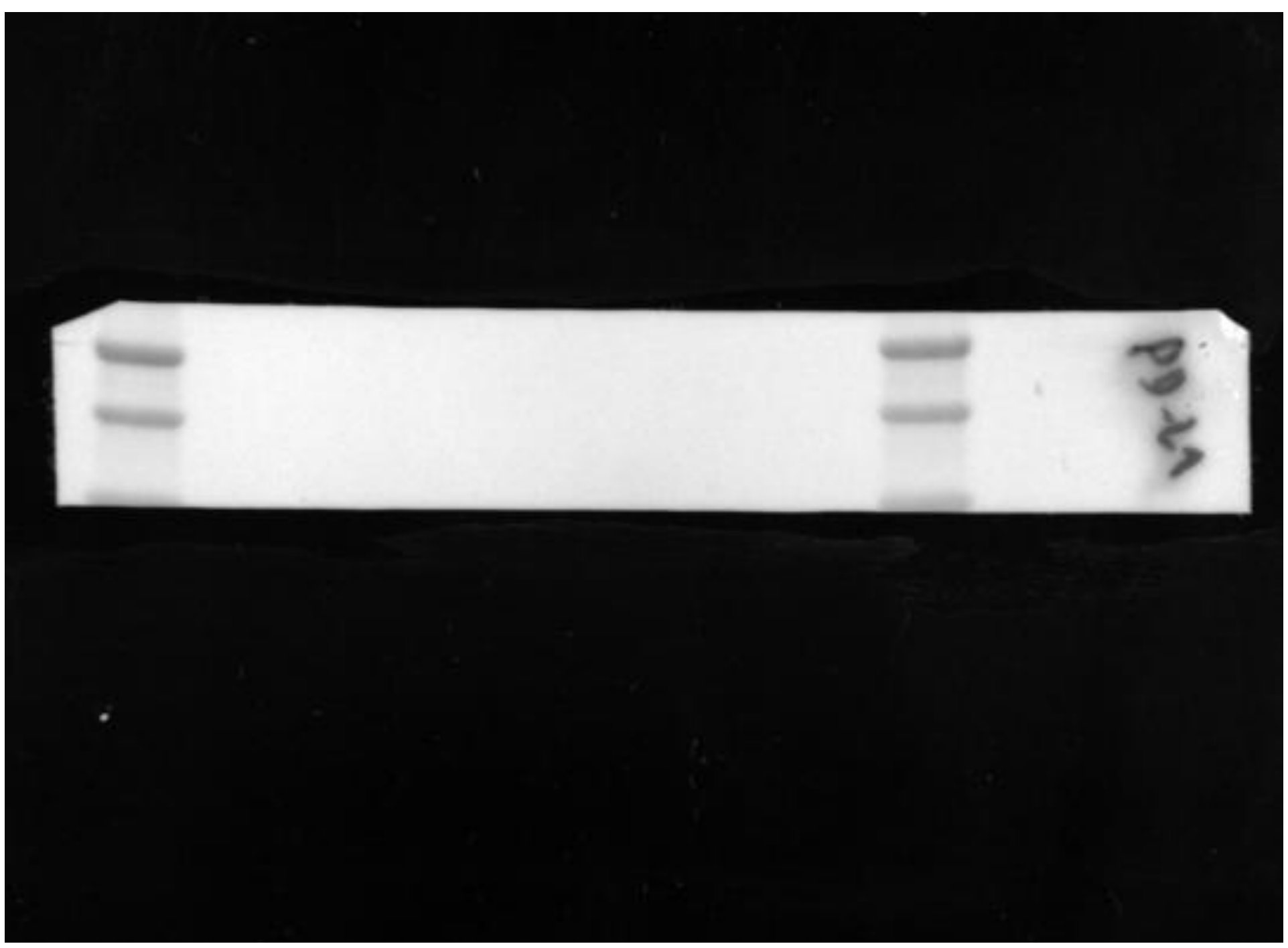

Phosphorylated STAT1

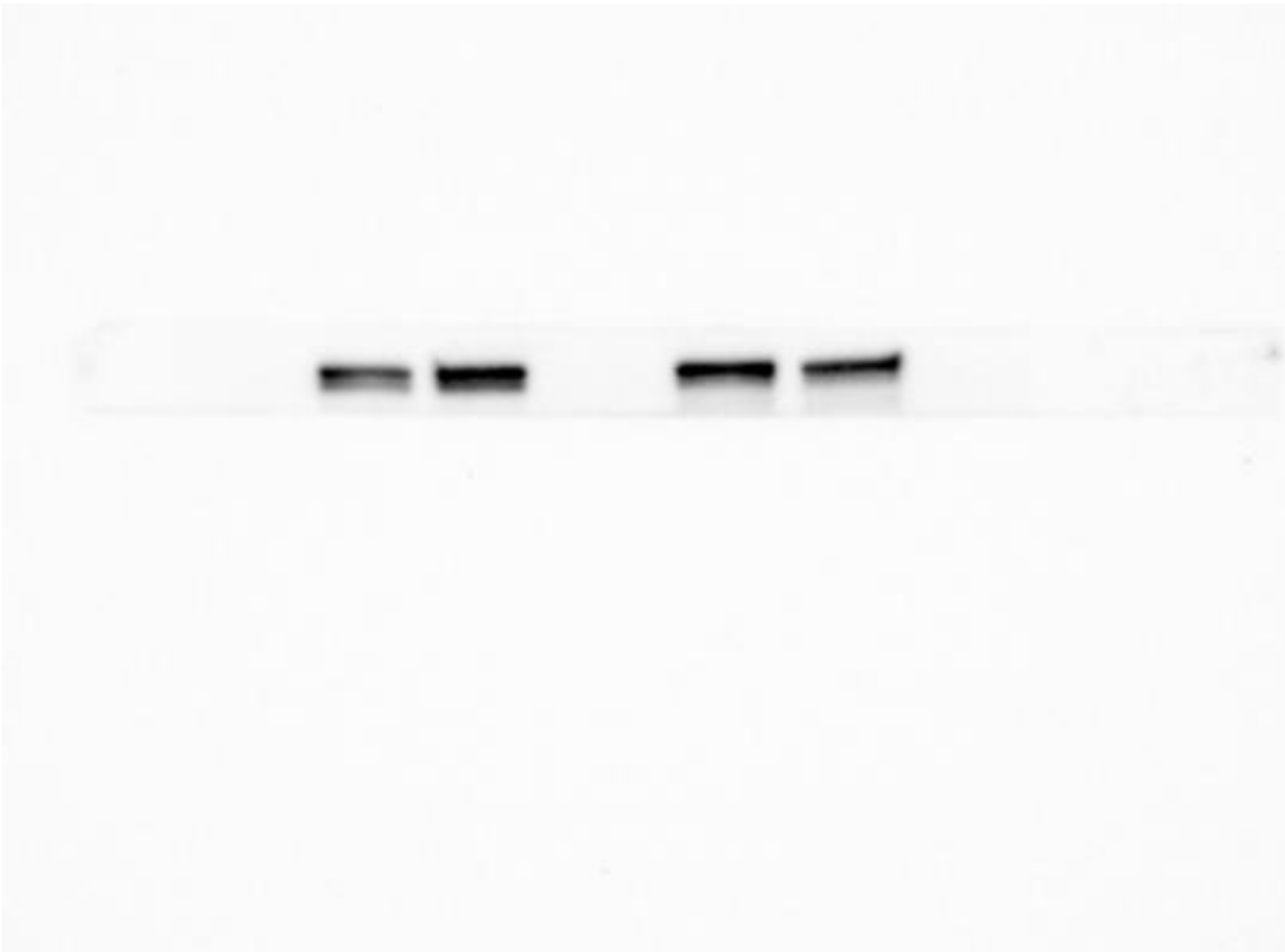

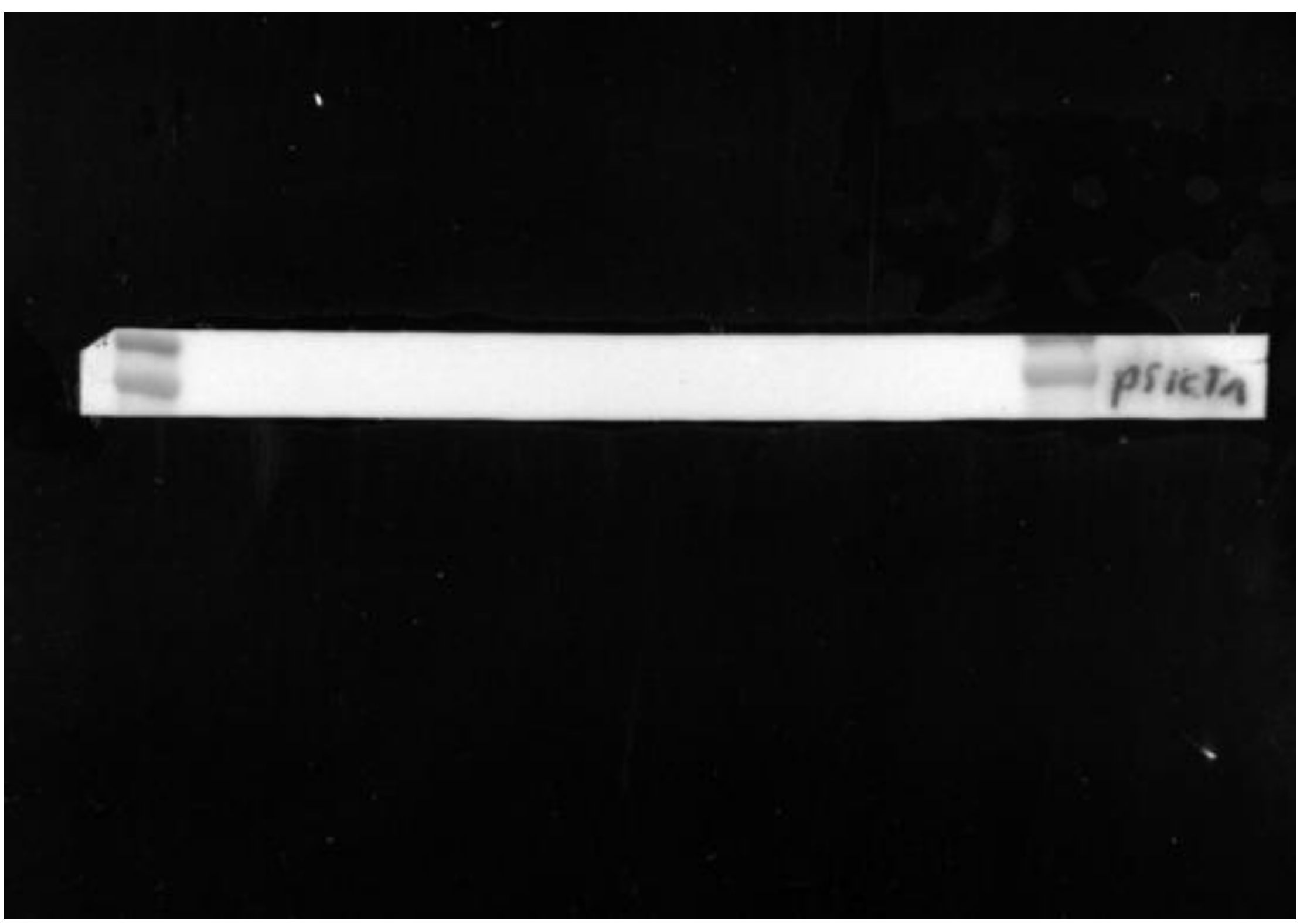

Total STAT1

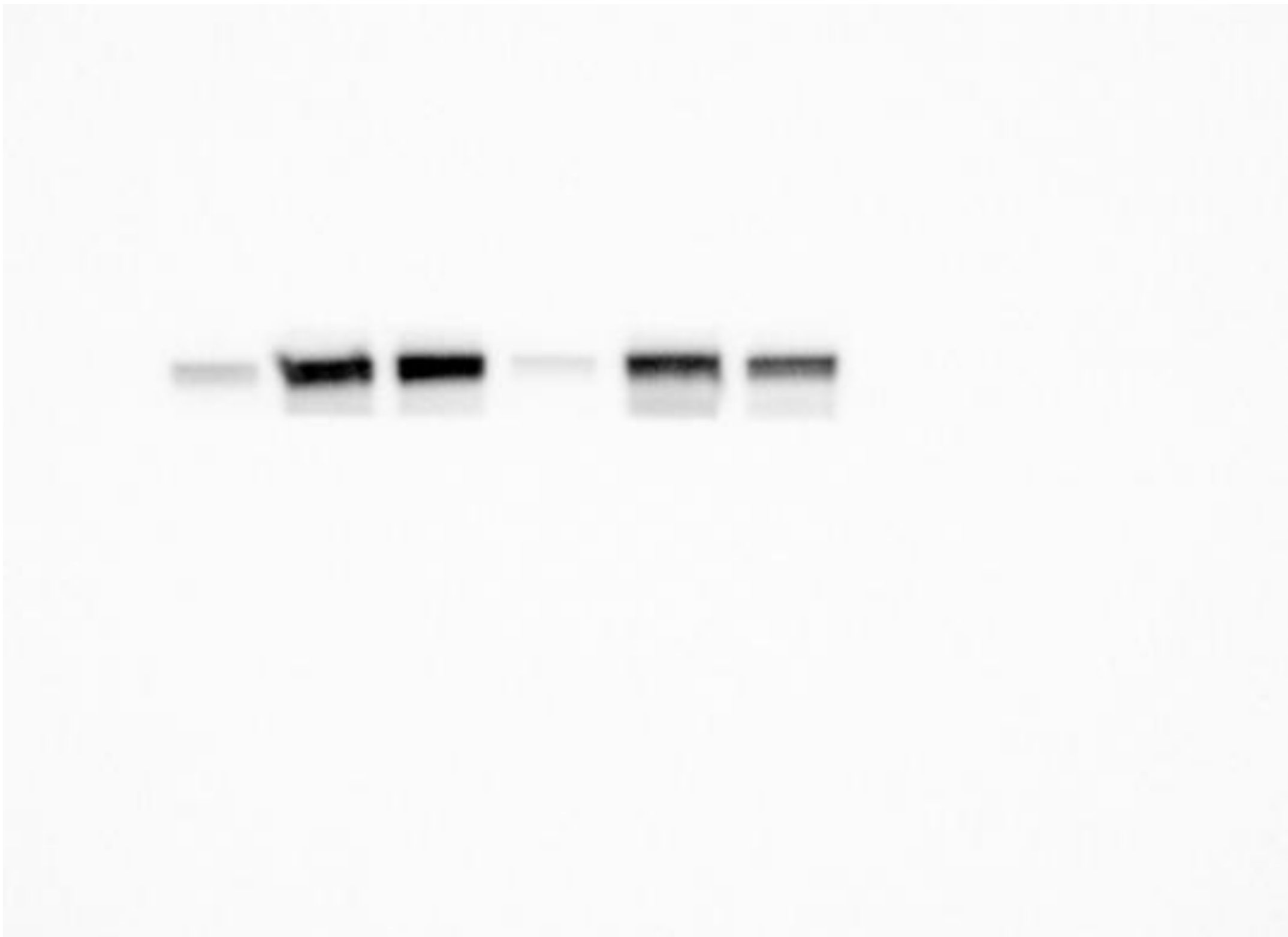

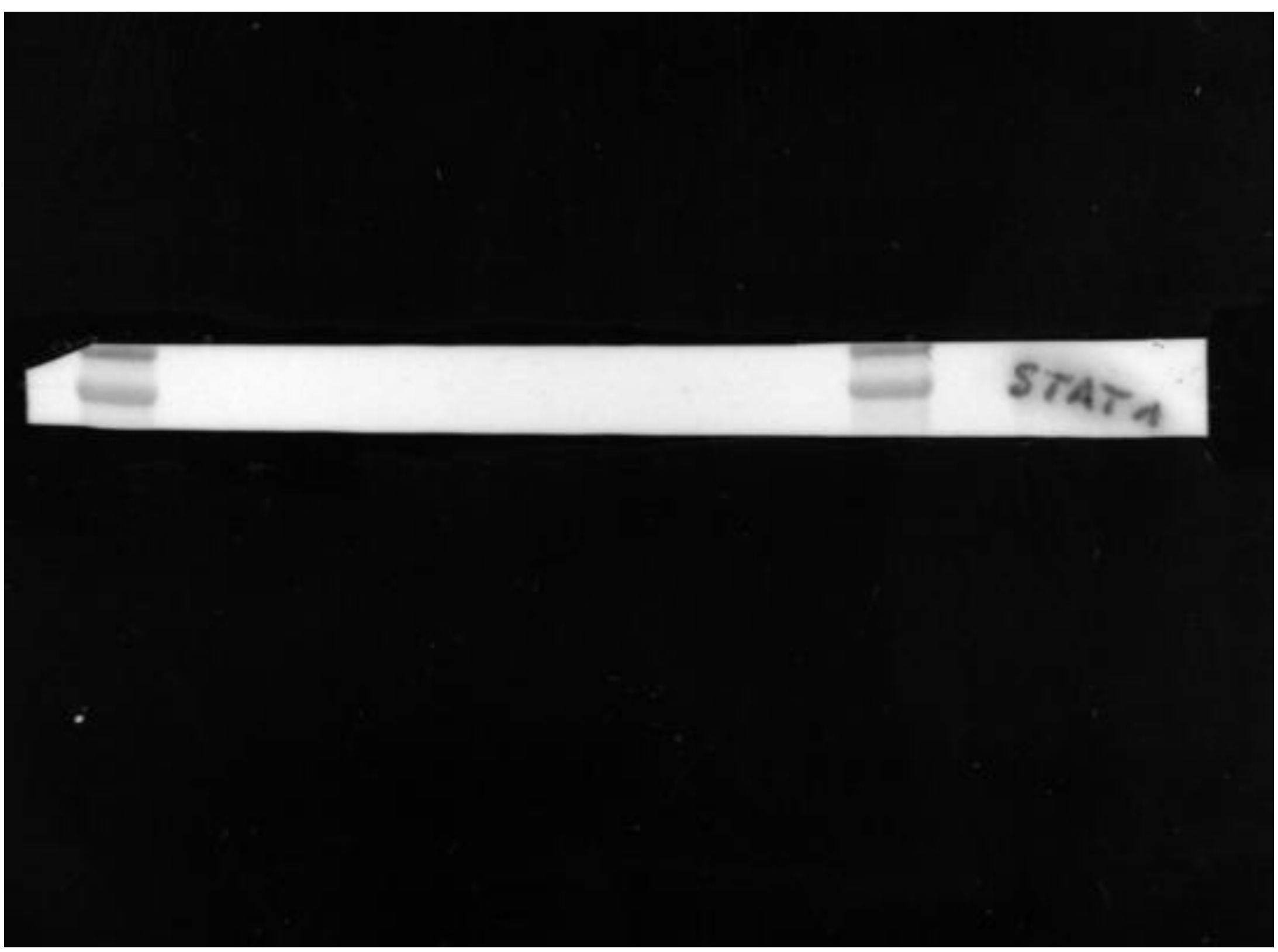

Vinculin

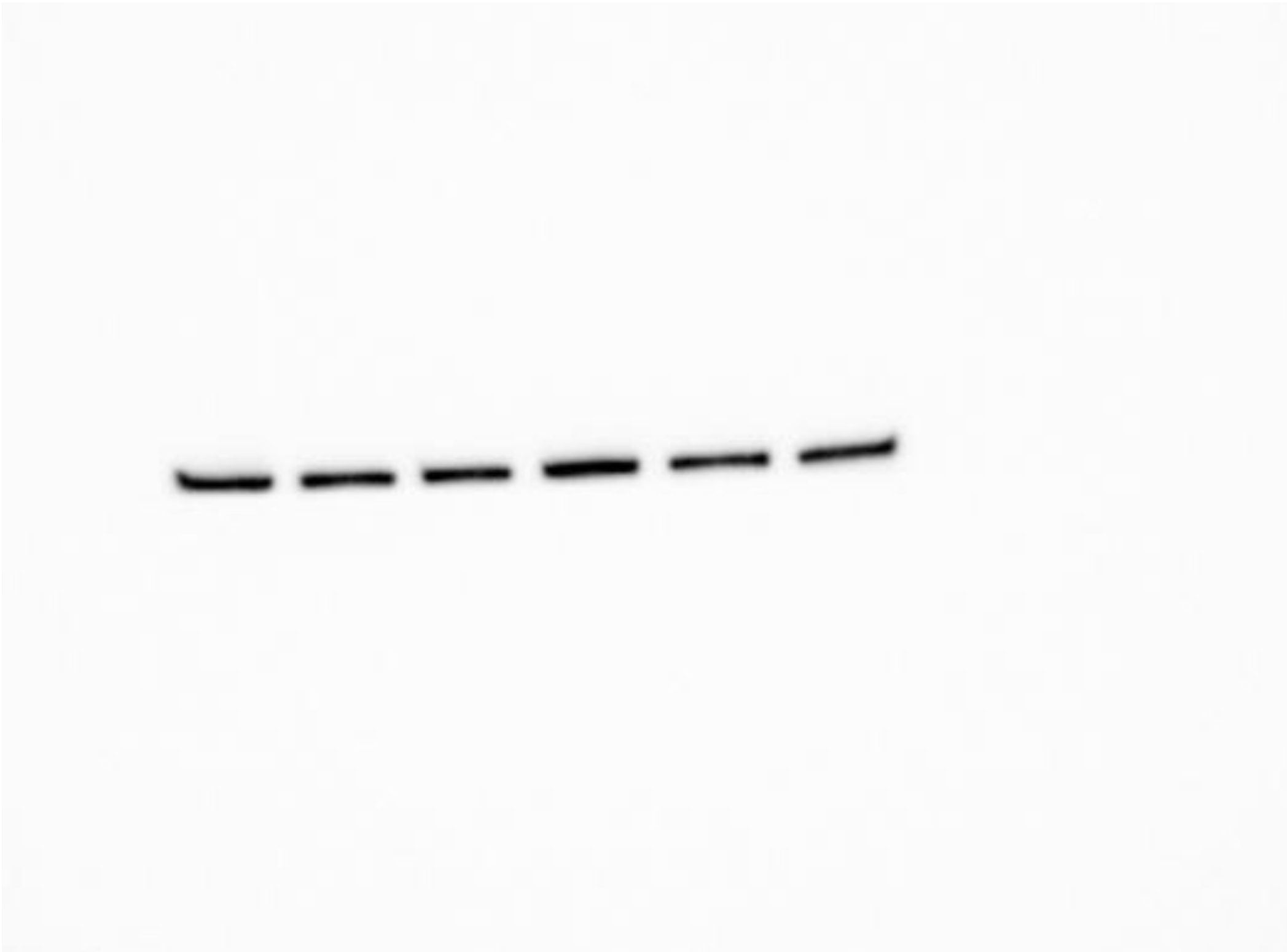

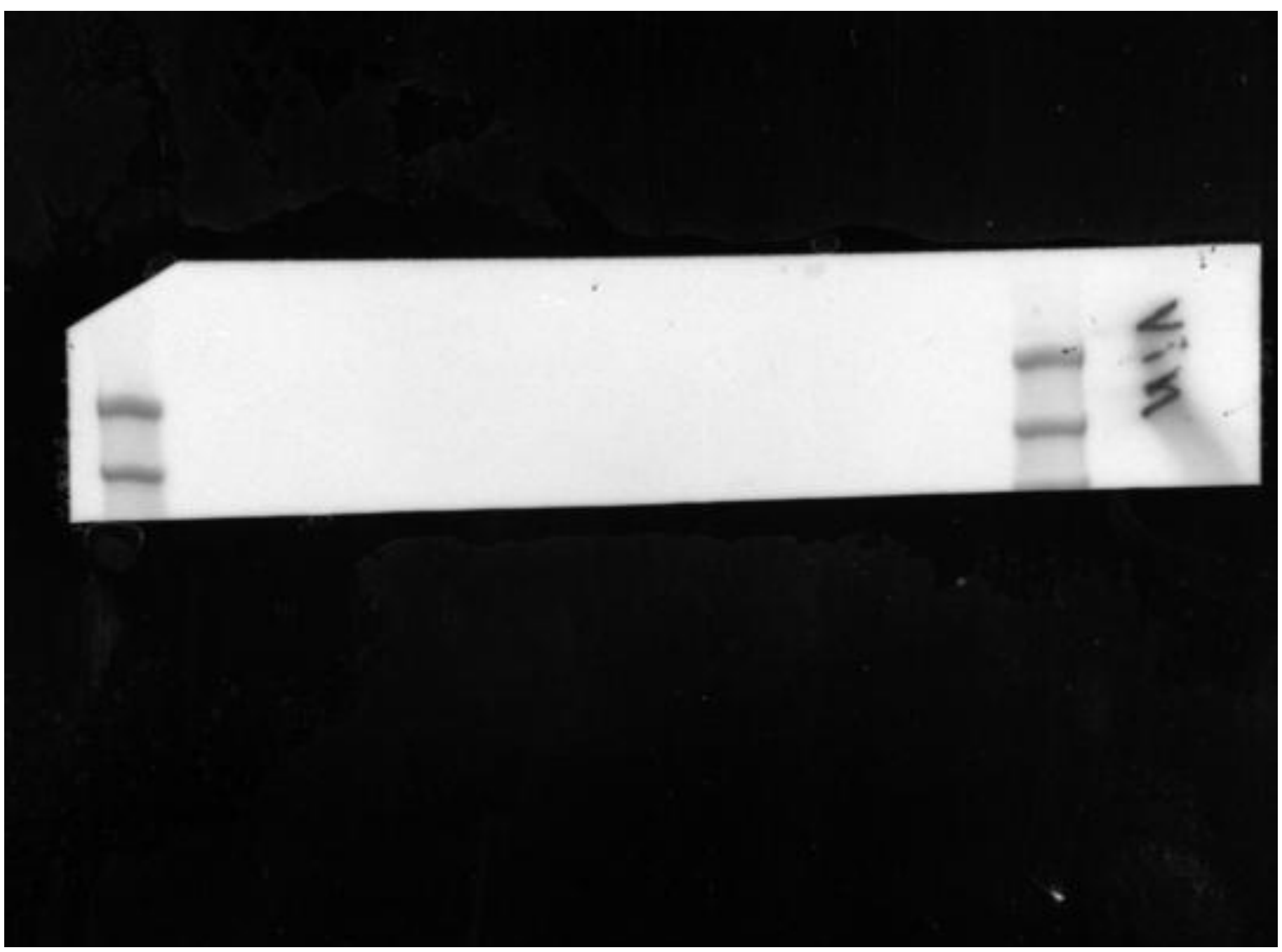

Supplement: Supplementary file 2 [file 12943_2026_2687_MOESM2_ESM.pdf]
